# The Safety of Multiple-Dose Liquid Blend Containing Kava and Kratom in Healthy Adults

**DOI:** 10.7759/cureus.75654

**Published:** 2024-12-13

**Authors:** Jaustin Dufour, Xinjie Lois Lin, Jun Wang, Joshua Baisley, Ambreen Atif, Erin C Berthold, Ramsey Atallah

**Affiliations:** 1 Medical Writing, Nutrasource Pharmaceutical and Nutraceutical Services Inc., Guelph, CAN; 2 Biostatistics, Nutrasource Pharmaceutical and Nutraceutical Services Inc., Guelph, CAN; 3 Clinical Trial Management, Nutrasource Pharmaceutical and Nutraceutical Services Inc., Guelph, CAN; 4 Consulting, Planted in Science Consulting, LLC, St. Augustine, USA; 5 Clinical Pharmacology, Botanic Tonics, LLC, Calabasas, USA

**Keywords:** kava, kratom, safety study, vital signs, withdrawal symptoms

## Abstract

This study investigates the safety of three different daily dosages of a liquid blend containing kava and kratom (Feel Free® Classic Tonic {FFCT}) in healthy adults over six consecutive days of supplementation. Both kava and kratom have been used traditionally for hundreds of years, but there is limited data on the combined safety of these ingredients. In this randomized, double-blind, placebo-controlled trial, the participants were assigned to receive one of three daily dosages of FFCT or placebo. Safety assessments included the monitoring of vital signs, clinical chemistry, hematology, and withdrawal symptoms using the Clinical Opiate Withdrawal Scale (COWS) and the Subjective Opiate Withdrawal Scale (SOWS). The results indicate that FFCT was safe, with mild to moderate adverse events (AEs) such as nausea, headaches, and fatigue, particularly in the high-dosage (HD) group. No significant changes in liver or kidney function were noted, and all vital signs remained within normal physiological ranges although some statistically significant changes in blood pressure (BP) and respiratory rate (RR) were observed. There were no clinically significant observations in COWS or SOWS scores despite a small but statistically significant increase in COWS total score in the high-dose group on day 7. Overall, FFCT appears safe for short-term use in healthy adults, with no significant impact on vital signs, laboratory values, or withdrawal symptoms.

## Introduction

Kava (*Piper methysticum*) is a plant native to the islands of the Pacific Ocean, which has been used for centuries by Polynesian cultures for its relaxing effects [1,2]. The traditional way of consuming kava is by making a drink from the roots of the plant [3]. The roots contain lactones (also known as kavalactones) that have been shown to have antianxiety effects, possibly by interacting with the gamma-aminobutyric acid receptors in the brain [4]. Two of the most commonly studied bioactive constituents of kava extracts are kavain and dihydrokavain [1]. A systematic review of published randomized controlled trials administering kava at dosages ranging from 60 to 280 mg kavalactones/day, for no longer than six months, reports that it is generally safe with the most frequent adverse events (AEs) being mild and transient [5].

Kratom (*Mitragyna speciosa*) is a tropical evergreen tree from the Rubiaceae family, native to Southeast Asia, and its leaves have been used as herbal remedies since at least the mid-19th century [6]. The bioactive alkaloids in kratom may have favorable interactions with neurotransmitter receptors in the central nervous system (D2 dopamine, 5-hydroxytryptamine 2A {HT2A}, and 5-HT2C receptors), which could confer potential benefits for anxiety and other mental health conditions [7,8]. Mitragynine stands as the primary alkaloid in kratom leaves, metabolized to form the active metabolite 7-hydroxymitragynine. A cross-sectional study compared hematology and clinical chemistry parameters between regular kratom users (daily dosages ranging from 76.3 to 114.8 mg of mitragynine) and healthy nonuser controls, demonstrating no significant differences [9].

Given their similar effects on anxiety and mood, kava and kratom are formulated into a liquid blend, Feel Free® Classic Tonic (FFCT). FFCT contains kava extract (standardized to 30% kavalactones) and kratom dried leaf powder. The safety of kava and kratom as individual ingredients has been established in previous studies [5,9]. However, there is no published data on the safety of a combination product containing both kava and kratom. This study addresses that gap by evaluating the short-term safety of FFCT in healthy adults.

The primary objective of this study is to assess the safety of FFCT for six consecutive days of supplementation at three different dosages, with a focus on vital signs, clinical laboratory parameters, and adverse events. In addition, the Clinical Opiate Withdrawal Scale (COWS) and Subjective Opiate Withdrawal Scale (SOWS) were used to monitor any signs of opioid-like withdrawal symptoms [10,11]. These scales are particularly relevant for kratom, which contains compounds that interact with opioid receptors, potentially leading to dependence or withdrawal symptoms upon cessation [12]. Respiratory rate (RR) and oxygen saturation (SpO_2_) were also monitored because opioids can cause respiratory depression. However, kratom has been shown to produce minimal respiratory depression with no sedative effects, unlike mu-opioid receptor agonists such as morphine [13].

Each dosing period in this study consists of six consecutive days of dosing, followed by a three-day follow-up period. During each period, the participants receive the designated dosage daily from day 1 to day 6, with follow-up visits conducted on day 7 and day 9 for safety assessments. In the first two periods, the participants receive either FFCT (low-dosage {LD} period) or placebo in a randomized, double-blind, crossover design. In the final two periods (mid-dosage {MD} and high-dosage {HD} periods), the participants received FFCT in an open-label design without a placebo. A 14-day washout period separated the placebo and low-dosage periods, while 28-day washouts separated the LD, MD, and HD periods.

## Materials and methods

Healthy adults 21-55 years of age (inclusive) with a body mass index (BMI) in the range of 18.5-29.9 kg/m^2^ (inclusive) who are naïve to kratom, or only occasionally use kratom, were eligible for inclusion in the study. Individuals were excluded if they had an abnormal respiratory rate (RR) or oxygen saturation (SpO_2_) result at screening or a history of heart disease, asthma, or cancer.

Each 15 mL of FFCT contains 410 mg kava extract (standardized to 30% kavalactones {123 mg}) and 840 mg kratom dried leaf powder (12.5 mg mitragynine and <0.05 mg 7-hydroxymitragynine). The main bioactive constituents of the kava extract are kavain and dihydrokavain, while the main bioactive constituent of the kratom leaf is mitragynine, which is metabolized to form the active metabolite 7-hydroxymitragynine.

For this multiple-dose escalation study, there were four dosing periods in total, with each dosing period including six consecutive days of dosing and a three-day follow-up period. The participants first received FFCT or placebo for six consecutive days at a dosage of 15 mL once per day in a randomized, double-blind, crossover manner (i.e., each participant received both FFCT and placebo, with each product tested in one of the two dosing periods). Between the placebo and low-dosage periods was a minimum 14-day washout period. Following that, higher dosage levels of FFCT were investigated in two more dosing periods of the same length in an open-label manner without a placebo; the two dosage levels include 15 mL twice per day six hours apart and 30 mL twice per day six hours apart for a total daily intake of 60 mL.

The FFCT dosage during the low-dose/placebo period is 15 mL (0.5 oz) per day, which provides 410 mg kava root extract, standardized to 30% kavalactones (126.3 mg total kavalactones) and 840 mg dried kratom leaf powder containing 12.5 mg mitragynine and less than 0.05 mg 7-hydroxymitragynine. During the mid-dose period, the dosage is 15 mL twice daily, equating to 30 mL (1.0 oz) per day. This provides a daily intake of 820 mg kava root extract, standardized to 30% kavalactones (252.6 mg total kavalactones), and 1680 mg dried kratom leaf powder containing 25 mg mitragynine and less than 0.05 mg 7-hydroxymitragynine. For the high-dose period, the dosage is 30 mL twice daily, resulting in a total of 60 mL (2.0 oz) per day. This provides 1640 mg kava root extract, standardized to 30% kavalactones (505.2 mg total kavalactones), and 3360 mg dried kratom leaf powder containing 50 mg mitragynine and less than 0.05 mg 7-hydroxymitragynine.

Between the low-dosage, mid-dosage, and high-dosage periods was a minimum 28-day washout period. A safety review including laboratory assessments, adverse events (AEs), and the assessment of COWS and SOWS was performed after each dosing period. After each safety review, a decision was made to proceed with the dosage escalation, and the participants who were eligible, willing, and able to proceed with the subsequent dosing period returned. The participants were replaced to maintain a sample size of 40 participants entering each dosing period (except for the last dosing period for the highest dosage level, which only required 35 participants to enter the dosing period). Each dosing period consisted of six consecutive in-clinic dosing days (day 1 to day 6), followed by two safety visits on day 7 and day 9.

To assess the safety of FFCT, vital signs (blood pressure {BP}, heart rate {HR}, RR, and SpO_2_), laboratory blood tests, adverse events, and COWS and SOWS were collected. BP, HR, laboratory blood tests, and adverse events were collected on day 1, day 6, day 7, and day 9. Anthropometrics and vital signs were collected at baseline (day 1) and day 6. SOWS and COWS were collected at baseline (day 1), day 7, and day 9.

Statistical analyses were performed using SAS 9.4 (SAS Institute Inc., Cary, NC). For within-group comparisons, paired t-tests were used for normally distributed data, while signed-rank tests were applied for non-normally distributed data. Similarly, between-group comparisons utilized independent t-tests for normally distributed data and Wilcoxon rank-sum tests for non-normally distributed data. Normality was assessed using the Shapiro-Wilk test, with significance evaluated at a two-sided alpha level of 0.05.

The Institutional Review Board of Advarra issued approval Pro00071515.

## Results

Participant disposition and demographics

**Figure 1:**
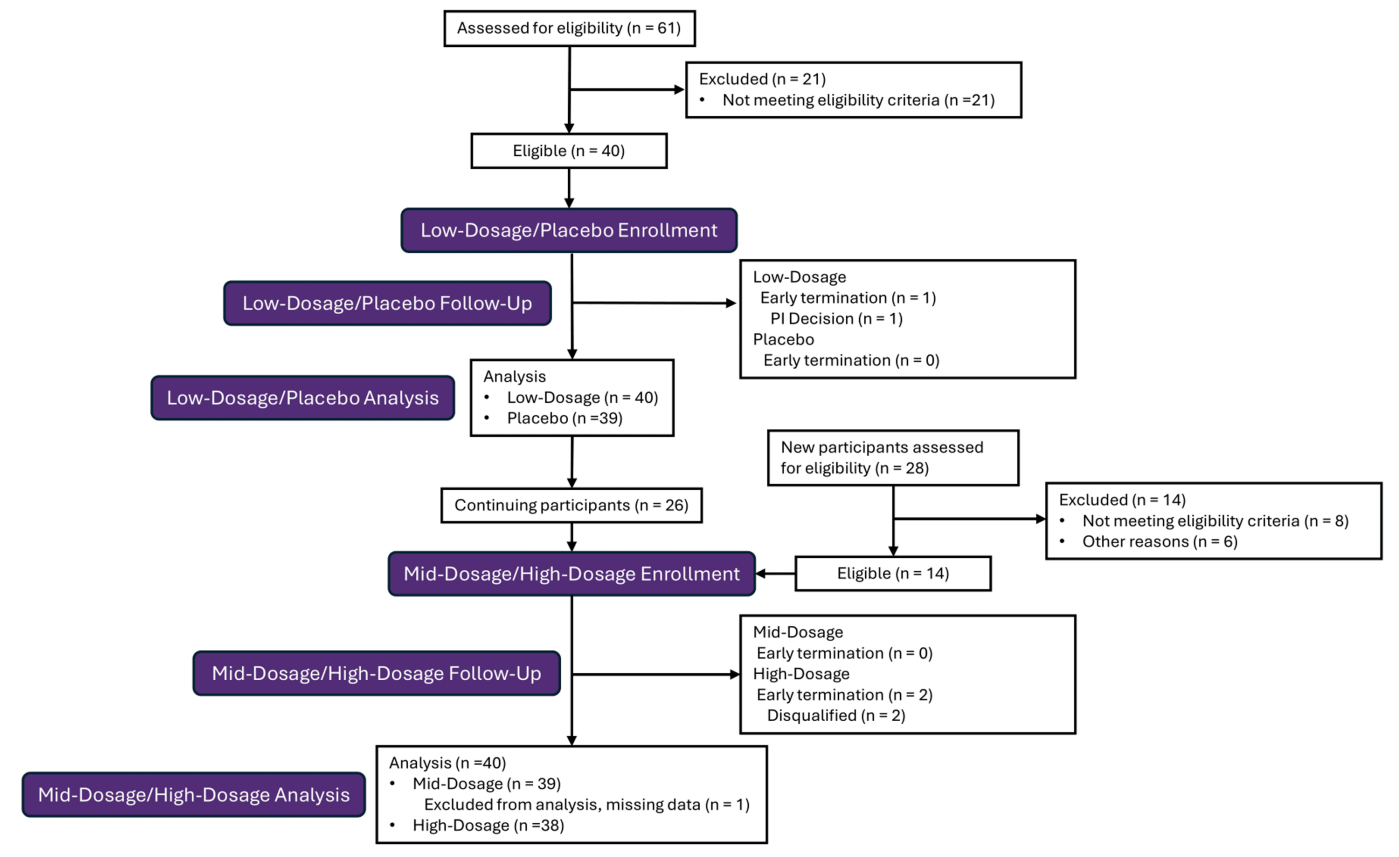
CONSORT Flow Diagram n, number of participants; PI, principal investigator; CONSORT, Consolidated Standards of Reporting Trials

Figure 1 presents the Consolidated Standards of Reporting Trials (CONSORT) flow diagram of the study, which was initiated in August 2023 and completed in September 2024. For the low-dosage/placebo periods, a total of 61 participants were screened for eligibility to obtain the planned sample size of 40 participants (21 participants screened failed). One participant was early terminated from the study by a physician's decision due to the participant's inability to comply with the study visit schedule. After a washout period of at least two months, 26 participants returned for the mid-dosage (MD) and high-dosage (HD) periods, requiring 28 participants to be screened to obtain 14 replacement participants for a total of 40 entering this open-label period (eight participants screened failed, and six were not randomized). During the HD period, two participants were disqualified. Similar to the full analysis set (FAS), there was a significant difference between low-dosage (LD) and placebo periods in diastolic and systolic blood pressures (P < 0.05), caused by the significant decrease with placebo (P < 0.05). In the HD period, similar to the FAS, there was a significant reduction in respiratory rate compared to baseline, which was also significant compared to placebo (P < 0.05). For all other parameters (weight, BMI, heart rate, and SpO_2_), no significant differences from placebo were observed (P > 0.05). None of the changes in vital signs were clinically significant. Clinical chemistry and hematology parameters were categorized as normal, low, or high at baseline and day 6. Overall, there was no consistent trend in the shifts of laboratory parameters.

There was no serious adverse events or death in this study. All adverse events resolved by the end of the study. In the placebo period, nine participants experienced a total of 14 adverse events, all of which were mild in severity. Of these adverse events, two were suspected to be related to the study product. These suspected to be related events were mildly elevated aspartate aminotransferase** **(AST) and alanine aminotransferase (ALT) in one participant, which was discussed above. In the LD period, six participants experienced a total of 17 adverse events, 14 of which were mild and three were moderate in severity. Of these adverse events, three were suspected to be related to the study product. These suspected to be related events were worsening fatigue and moderate severity, which occurred in one participant on the second, third, and fourth days of dosing. Each event lasted about two hours before resolution. In the MD period, 10 participants experienced a total of 18 adverse events, all of which were mild in severity. Of these adverse events, 13 were suspected to be related to the study product. The most common events were headaches in three participants (7.5% of all participants) and decreased appetite in two participants (5%). In the HD period, 17 participants experienced a total of 90 adverse events, five of which were moderate in severity, and the remaining 85 events were mild. Of these adverse events, 86 were suspected to be related to the study product. The most common events were nausea in 11 participants (28.9%), fatigue in eight participants (21.1%), decreased appetite in four participants (10.5%), headache in 11 participants (28.9%), and pruritus in five participants (13.2%).

Two sets of participants were identified; the first set included all participants who have received at least one dose of the study product and one post-dose assessment (hereinafter referred to as the full analysis set {FAS}), and the second set is a subset of participants, including those that had undergone the crossover (LD and placebo periods) study period, as well as the MD and HD periods (hereinafter referred to as the sub-group).

The average age of the participants at the beginning of the study was 39.8 ± 10.82 years (see Table 1) with an average body mass index (BMI) of 24.9 ± 3.08 kg/m^2^ (see Table 2) at the time of randomization. The majority of the participants were White (n = 24, 60.0%), followed by Asian (n = 6, 15.0%), Black or African American (n = 4, 10.0%), and multiple (n = 1, 2.5%); the remainder did not report their race (n = 5, 12.5%). There was an approximately even split between female (n = 19, 47.5%) and male (n = 21, 52.5%) participants. The demographics of the sub-group of participants were similar.

**Table 1:** Participant Demographics Data are presented as mean ± SD or n (%) ^a^Sub-group of the participants who had undergone the crossover (low-dosage and placebo) study period, as well as the mid- and high-dosage study period N: number of participants

		Analysis Sets	
Demographic		All Participants (N = 40)	Sub-group^a^ (N = 25)
Age (year)		39.8 ± 10.82	42.6 ± 11.11
Sex	Female	21 (52.5%)	15 (60.0%)
	Male	19 (47.5%)	10 (40.0%)
Race	Asian	6 (15.0%)	4 (16.0%)
	Black or African American	4 (10.0%)	3 (12.0%)
	White	24 (60.0%)	15 (60.0%)
	Multiple	1 (2.5%)	1 (4.0%)
	Not reported	5 (12.5%)	2 (8.0%)
Ethnicity	Hispanic or Latino	6 (15.0%)	3 (12.0%)
	Not Hispanic or Latino	34 (85.0%)	22 (88.0%)

**Table 2:** Change From Baseline to Day 6 in Anthropometrics and Vital Signs (FAS) Values are mean ± SD. At baseline, n = 39, 40, 40, and 38 for placebo, low-dosage (LD), mid-dosage (MD), and high-dosage (HD) groups, respectively. At day 6, n = 39, 40, 39, and 38 for placebo, low-dosage, mid-dosage, and high-dosage groups, respectively †A significant between-group difference compared to placebo (P < 0.05) *Significant within-group change from baseline to day 6 (P < 0.05) BMI, body mass index; FFCT, Feel Free® Classic Tonic; SpO_2_, oxygen saturation; Δ, change from baseline; FAS, full analysis set

			FFCT Dosage Levels			
Anthropometrics and Vital Signs	Study Visit	Placebo Period	LD Period (15 mL)	MD Period (15 mL + 15 mL)	HD Period (30 mL + 30 mL)	
						Weight (kg)	Baseline	72.31 ± 12.400	72.54 ± 12.270	73.20 ± 11.120	72.82 ± 11.006	
Day 6	72.43 ± 12.437	72.81 ± 12.396	73.38 ± 11.362	73.10 ± 10.797		
Δ	0.12 ± 0.833	0.27 ± 0.873	0.27 ± 1.288	0.28 ± 1.108		
BMI (kg/m^2^)	Baseline	24.93 ± 3.107	24.99 ± 3.071	25.27 ± 2.988	25.33 ± 2.889	
	Day 6	24.97 ± 3.110	25.08 ± 3.091	25.41 ± 3.031	25.41 ± 2.945	
	Δ	0.04 ± 0.284	0.10 ± 0.300	0.10 ± 0.443	0.08 ± 0.324	
Diastolic blood pressures (mmHg)	Baseline	73.4 ± 6.96	71.1 ± 6.71	75.2 ± 7.87	73.9 ± 8.55	
	Day 6	71.4 ± 7.30	72.2 ± 7.38	73.4 ± 8.65	73.5 ± 9.05	
	Δ	-2.0 ± 4.67*	1.1 ± 6.28†	-1.7 ± 7.95*	-0.4 ± 5.14	
Systolic blood pressure (mmHg)	Baseline	113.8 ± 11.90	111.8 ± 10.58	114.8 ± 12.46	113.5 ± 14.36	
	Day 6	110.6 ± 10.76	112.2 ± 11.38	112.7 ± 14.62	112.2 ± 11.83	
	Δ	-3.2 ± 6.13*	0.5 ± 6.57†	-1.9 ± 14.18	-1.2 ± 8.75	
Heart rate (beats/minute)	Baseline	69.2 ± 13.01	69.0 ± 10.99	70.7 ± 12.56	66.5 ± 11.97	
	Day 6	68.3 ± 10.57	68.3 ± 12.23	71.2 ± 12.31	69.8 ± 9.38	
	Δ	-0.9 ± 10.60	-0.7 ± 11.16	1.1 ± 10.29	3.3 ± 9.89*	
SpO_2_ (%)	Baseline	99.2 ± 1.06	98.7 ± 1.36	99.0 ± 1.05	99.0 ± 1.01	
	Day 6	98.8 ± 1.40	98.9 ± 1.31	98.5 ± 1.31	98.9 ± 1.21	
	Δ	-0.4 ± 1.07*	0.2 ± 1.29	-0.5 ± 1.07*	-0.1 ± 1.18	
Respiratory rate (breaths/minute)	Baseline	15.0 ± 2.55	15.5 ± 2.25	15.6 ± 2.18	15.2 ± 2.44	
	Day 6	15.5 ± 2.28	15.2 ± 2.43	15.0 ± 1.99	14.4 ± 2.55	
	Δ	0.5 ± 2.78	-0.3 ± 3.06	-0.5 ± 2.78	-0.8 ± 3.00†	

Anthropometrics and vital signs

There was a significant difference between LD and placebo periods for diastolic and systolic blood pressure (P < 0.05, Table 2), which was caused by significant decreases from baseline with placebo (P < 0.05) and a nonsignificant increase from baseline in LD (P > 0.05). In the HD period, there was a small but statistically significant reduction in respiratory rate compared to the placebo (P < 0.05). For all other parameters (weight, BMI, heart rate, and SpO_2_), no significant differences from placebo were observed (P > 0.05). None of the changes in vital signs were clinically significant.

An analysis of the sub-group is presented in Table 3. Similar to the FAS, there was a significant difference between LD and placebo in diastolic and systolic blood pressures (P < 0.05), caused by the significant decrease with placebo (P < 0.05). In the HD period, similar to the FAS, there was a significant reduction in respiratory rate compared to baseline, which was also significant compared to placebo (P < 0.05). For all other parameters (weight, BMI, heart rate, and SpO_2_), no significant differences from placebo were observed (P > 0.05). None of the changes in vital signs were clinically significant.

**Table 3:** Change From Baseline to Day 6 in Anthropometrics and Vital Signs (Sub-group Analysis) Values are mean ± SD. At baseline and day 6, n = 25, 25, 25, and 25 for placebo, low-dosage (LD), mid-dosage (MD), and high-dosage (HD) groups, respectively †A significant between-group difference compared to placebo (P < 0.05) *Significant within-group change from baseline to day 6 (P < 0.05) BMI, body mass index; FFCT, Feel Free® Classic Tonic; SpO_2_, oxygen saturation; Δ, change from baseline

			FFCT Dosage Levels		
Anthropometrics and Vital Signs	Study Visit	Placebo Period	LD Period (15 mL)	MD Period (15 mL + 15 mL)	HD Period (30 mL + 30 mL)
Weight (kg)	Baseline	73.03 ± 12.299	73.11 ± 12.372	72.88 ± 11.278	73.03 ± 11.465
	Day 6	73.15 ± 12.317	73.43 ± 12.353	73.15 ± 11.518	73.24 ± 11.447
	Δ	0.12 ± 0.924	0.32 ± 0.772*	0.26 ± 1.394	0.21 ± 1.034
BMI (kg/m^2^)	Baseline	25.39 ± 3.216	25.40 ± 3.188	25.35 ± 2.938	25.43 ± 2.901
	Day 6	25.42 ± 3.185	25.52 ± 3.200	25.44 ± 2.933	25.46 ± 2.927
	Δ	0.03 ± 0.313	0.12 ± 0.276*	0.09 ± 0.477	0.04 ± 0.277
Diastolic blood pressure (mmHg)	Baseline	75.2 ± 7.26	71.0 ± 6.11	75.1 ± 6.72	74.5 ± 7.78
	Day 6	72.7 ± 7.99	72.8 ± 7.60	73.6 ± 8.98	74.4 ± 7.69
	Δ	-2.5 ± 4.99*	1.8 ± 6.17†	-1.6 ± 8.89*	-0.1 ± 4.88
Systolic blood pressure (mmHg)	Baseline	115.8 ± 12.71	113.4 ± 11.07	113.9 ± 12.82	115.0 ± 15.20
	Day 6	111.5 ± 11.14	112.9 ± 11.67	113.6 ± 16.34	113.5 ± 11.74
	Δ	-4.2 ± 6.11*	-0.5 ± 6.17†	-0.3 ± 15.78	-1.5 ± 9.18
Heart rate (beats/minute)	Baseline	71.1 ± 12.07	69.4 ± 10.91	70.7 ± 13.89	67.4 ± 12.87
	Day 6	69.5 ± 9.15	69.5 ± 11.96	69.4 ± 12.08	69.6 ± 9.04
	Δ	-1.6 ± 11.96	0.1 ± 13.09	-1.4 ± 10.45	2.3 ± 9.19
SpO_2_ (%)	Baseline	99.2 ± 0.93	99.0 ± 1.12	99.1 ± 1.08	99.0 ± 0.98
	Day 6	98.8 ± 1.36	99.1 ± 1.12	98.6 ± 1.39	99.0 ± 1.21
	Δ	-0.5 ± 1.26	0.1 ± 1.19	-0.5 ± 1.00*	0.0 ± 1.15
Respiratory rate (breaths/minute)	Baseline	14.6 ± 2.27	15.2 ± 2.31	15.5 ± 2.10	15.1 ± 2.59
	Day 6	15.2 ± 2.31	15.0 ± 2.39	15.0 ± 1.74	13.6 ± 2.00
	Δ	0.6 ± 2.75	-0.2 ± 2.94	-0.5 ± 2.66	-1.5 ± 2.90†*

Clinical chemistry and hematology

Clinical chemistry (see Table 4 and Appendices) and hematology parameters (see Appendices) were categorized as normal, low, or high at baseline and day 6. Overall, there was no consistent trend in the shifts of laboratory parameters. See Appendices for a summary of the sub-group analysis for clinical chemistry and hematology.

**Table 4:** Clinical Chemistry Test Shift From Baseline to Day 6 (FAS) At baseline, n = 39, 40, 40, and 38 for placebo, low-dosage (LD), mid-dosage (MD), and high-dosage (HD) groups, respectively. At day 6, n = 39, 40, 39, and 38 for placebo, low-dosage, mid-dosage, and high-dosage groups, respectively. For a full summary of clinical chemistry test shifts, see Appendices ALT, alanine aminotransferase (U/L); AST, aspartate aminotransferase (U/L); eGFR, estimated glomerular filtration rate (mL/minute/1.73 m^2^); FFCT, Feel Free® Classic Tonic; TB, total bilirubin (umol/L); Δ, change from baseline; FAS, full analysis set

						FFCT Dosage Levels									
			Placebo Period Baseline			LD Period (15 mL) Baseline			MD Period (15 mL + 15 mL) Baseline			HD Period (30 mL + 30 mL) Baseline			
Clinical Chemistry Parameter	Study Visit	Status	Low	Normal	High	Low	Normal	High	Low	Normal	High	Low	Normal	High	
															ALT	Day 6	Low	0 (0%)	0 (0%)	0 (0%)	0 (0%)	0 (0%)	0 (0%)	0 (0%)	0 (0%)	0 (0%)	0 (0%)	0 (0%)	0 (0%)	
Normal	0 (0%)	36 (92.3%)	1 (2.6%)	0 (0%)	38 (95.0%)	1 (2.5%)	0 (0%)	39 (97.5%)	0 (0%)	0 (0%)	37 (97.4%)	0 (0%)				
High	0 (0%)	1 (2.6%)	1 (2.6%)	0 (0%)	0 (0%)	1 (2.5%)	0 (0%)	0 (0%)	0 (0%)	0 (0%)	0 (0%)	1 (2.6%)				
													AST	Day 6			Low	0 (0%)	0 (0%)	0 (0%)	0 (0%)	0 (0%)	0 (0%)	0 (0%)	0 (0%)	0 (0%)	0 (0%)	0 (0%)	0 (0%)	
Normal	0 (0%)	36 (92.3%)	2 (5.1%)	0 (0%)	39 (97.5%)	1 (2.5%)	0 (0%)	38 (95.0%)	1 (2.5%)	0 (0%)	37 (97.4%)	0 (0%)			
High	0 (0%)	1 (2.6%)	0 (0%)	0 (0%)	0 (0%)	0 (0%)	0 (0%)	0 (0%)	0 (0%)	0 (0%)	0 (0%)	1 (2.6%)			
															TB	Day 6	Low	0 (0%)	0 (0%)	0 (0%)	0 (0%)	0 (0%)	0 (0%)	0 (0%)	1 (2.5%)	0 (0%)	0 (0%)	0 (0%)	0 (0%)	
Normal	0 (0%)	37 (94.9%)	2 (5.1%)	0 (0%)	38 (95.0%)	0 (0%)	0 (0%)	37 (92.5%)	1 (2.5%)	0 (0%)	37 (97.4%)	0 (0%)				
High	0 (0%)	0 (0%)	0 (0%)	0 (0%)	1 (2.5%)	1 (2.5%)	0 (0%)	0 (0%)	0 (0%)	0 (0%)	1 (2.6%)	0 (0%)				
													eGFR	Day 6			Low	0 (0%)	0 (0%)	0 (0%)	0 (0%)	0 (0%)	0 (0%)	0 (0%)	0 (0%)	0 (0%)	0 (0%)	0 (0%)	0 (0%)	
Normal	0 (0%)	39 (100%)	0 (0%)	0 (0%)	40 (100%)	0 (0%)	1 (2.5%)	38 (95.0%)	0 (0%)	0 (0%)	38 (100%)	0 (0%)			
High	0 (0%)	0 (0%)	0 (0%)	0 (0%)	0 (0%)	0 (0%)	0 (0%)	0 (0%)	0 (0%)	0 (0%)	0 (0%)	0 (0%)			

Withdrawal symptoms (COWS and SOWS)

The following score ranges apply for the Clinical Opiate Withdrawal Scale (COWS): A score of 5-12 indicates mild withdrawal, 13-24 indicates moderate withdrawal, 25-36 indicates moderately severe withdrawal, and more than 36 indicates severe withdrawal [11]. A COWS score of less than 5 is indicative of no withdrawal. Throughout the study, all recorded COWS scores were below 5, indicating no clinically significant withdrawal symptoms were observed at any point.

A statistically significant increase from baseline in resting pulse rate was observed in the HD period on day 7, which was also significantly different from placebo (P < 0.05, Appendices). No significant between-group differences were observed for pulse rate changes in the LD or MD periods (P > 0.05). For all other COWS parameters, including sweating, restlessness, pupil size, bone or joint aches, runny nose or tearing, gastrointestinal upset, tremor, yawning, anxiety or irritability, gooseflesh skin, and respiratory rate, no significant differences from placebo or baseline were observed (P > 0.05). For the total COWS score, the HD period showed a small but significant increase compared to placebo on day 7; this was also a significant increase from baseline (P < 0.05). No other period exhibited significant differences in the total COWS score compared to placebo (P > 0.05, Table 5).

**Table 5:** Change From Baseline to Day 6 in COWS and SOWS Total Scores (FAS) Values are mean ± SD. At baseline, n = 39, 40, 40, and 38 for placebo, low-dosage (LD), mid-dosage (MD), and high-dosage (HD) groups, respectively. At day 7 and day 9, n = 39, 39, 40, and 38 for placebo, low-dosage, mid-dosage, and high-dosage groups, respectively †A significant between-group difference compared to placebo (P < 0.05) *Significant within-group change from baseline to day 6 (P < 0.05) FAS, full analysis set; FFCT, Feel Free® Classic Tonic; COWS, Clinical Opiate Withdrawal Scale; SOWS, Subjective Opiate Withdrawal Scale; Δ, change from baseline

			FFCT Dosage Levels		
COWS and SOWS Total Scores	Study Visit	Placebo Period	LD Period (15 mL)	MD Period (15 mL + 15 mL)	HD Period (30 mL + 30 mL)
COWS total score	Baseline	0.0 ± 0.16	0.0 ± 0.00	0.2 ± 0.36	0.1 ± 0.23
	Day 7	0.1 ± 0.27	0.1 ± 0.41	0.3 ± 0.76	0.6 ± 0.98
	Δ	0.1 ± 0.32	0.1 ± 0.41	0.2 ± 0.74	0.5 ± 1.01†*
	Day 9	0.1 ± 0.22	0.0 ± 0.16	0.2 ± 0.61	0.2 ± 0.49
	Δ	0.0 ± 0.16	0.0 ± 0.16	0.1 ± 0.68	0.2 ± 0.51
SOWS total score	Baseline	0.3 ± 1.28	0.0 ± 0.00	0.0 ± 0.00	0.0 ± 0.00
	Day 7	0.1 ± 0.38	0.3 ± 1.36	0.3 ± 1.16	0.3 ± 1.03
	Δ	-0.2 ± 1.19	0.3 ± 1.36	0.3 ± 1.16	0.3 ± 1.03
	Day 9	0.0 ± 0.00	0.1 ± 0.80	0.2 ± 0.95	0.0 ± 0.00
	Δ	-0.3 ± 1.28	0.1 ± 0.80	0.2 ± 0.95	0.0 ± 0.00

There were no statistically significant differences from placebo for any FFCT dosage period in total SOWS score (P > 0.05, Appendices). No significant differences between placebo and any of the dosage periods were observed for SOWS scores, including anxiousness, bone and muscle aches, restlessness, nausea, vomiting, muscle twitching, stomach cramps, feeling like shooting up, yawning, perspiration, eyes tearing, runny nose, gooseflesh, shaking, hot flashes, or cold flashes (P > 0.05). For a full summary of COWS and SOWS scores, see Appendices.

An analysis of a sub-group is presented in Appendices for COWS and SOWS. Similar to the FAS results, for the COWS total score, there was a statistically significant increase from baseline in the high-dosage group on day 7 compared to baseline, which was also significant compared to placebo (P < 0.05). The average COWS scores remained well below the cutoff for mild withdrawal (<5), indicating that no participants in this study were classified as experiencing withdrawal, either clinically or subjectively.

Adverse events

There was no serious adverse events or death in this study. All adverse events resolved by the end of the study. See Table 6 for a summary of adverse events by relation and severity and Appendices for a summary of adverse events by system organ class and preferred term.

**Table 6:** Adverse Events FFCT, Feel Free® Classic Tonic; HD, high dosage; LD, low dosage; MD, mid-dosage; N, number of participants

				FFCT Dosage Levels					
		Placebo Period		LD Period (15 mL)		MD Period (15 mL + 15 mL)		HD Period (30 mL + 30 mL)	
Adverse Events		Participants (N = 39)	Events (N = 14)	Participants (N = 40)	Events (N = 17)	Participants (N = 40)	Events (N = 18)	Participants (N = 38)	Events (N = 90)
Overall		9 (23.1%)	14 (100%)	6 (15.0%)	17 (100%)	10 (25.0%)	18 (100%)	17 (44.7%)	90 (100%)
Relation	Related	0 (0%)	0 (0%)	0 (0%)	0 (0%)	0 (0%)	0 (0%)	0 (0%)	0 (0%)
	Suspected	1 (2.6%)	2 (14.3%)	1 (2.5%)	3 (17.6%)	8 (20.0%)	13 (72.2%)	17 (44.7%)	86 (95.6%)
	Not related	8 (20.5%)	12 (85.7%)	6 (15.0%)	14 (82.4%)	4 (10.0%)	5 (27.8%)	3 (7.9%)	4 (4.4%)
Severity	Severe	0 (0%)	0 (0%)	0 (0%)	0 (0%)	0 (0%)	0 (0%)	0 (0%)	0 (0%)
	Moderate	0 (0%)	0 (0%)	2 (5.0%)	4 (23.5%)	0 (0%)	0 (0%)	4 (10.5%)	5 (5.6%)
	Mild	9 (23.1%)	14 (100%)	6 (15.0%)	13 (76.5%)	10 (25.0%)	18 (100%)	17 (44.7%)	85 (94.4%)
Serious		0 (0%)	0 (0%)	0 (0%)	0 (0%)	0 (0%)	0 (0%)	0 (0%)	0 (0%)
Leading to discontinuation		0 (0%)	0 (0%)	0 (0%)	0 (0%)	0 (0%)	0 (0%)	0 (0%)	0 (0%)

In the placebo period, nine participants experienced a total of 14 adverse events, all of which were mild in severity. Of these adverse events, two were suspected to be related to the study product. These suspected to be related events were mild elevated AST and ALT in one participant, which was discussed above.

In the LD period, six participants experienced a total of 17 adverse events, 14 of which were mild and three were moderate in severity. Of these adverse events, three were suspected to be related to the study product. These suspected to be related events were worsening fatigue, moderate severity, which occurred in one participant on the second, third, and fourth days of dosing. Each event lasted about two hours before resolution.

In the MD period, 10 participants experienced a total of 18 adverse events, all of which were mild in severity. Of these adverse events, 13 were suspected to be related to the study product. The most common events were headaches in three participants (7.5% of all participants) and decreased appetite in two participants (5%).

In the HD period, 17 participants experienced a total of 90 adverse events, five of which were moderate in severity, and the remaining 85 events were mild. Of these adverse events, 86 were suspected to be related to the study product. The most common events were nausea in 11 participants (28.9%), fatigue in eight participants (21.1%), decreased appetite in four participants (10.5%), headache in 11 participants (28.9%), and pruritus in five participants (13.2%).

## Discussion

The purpose of this study was to evaluate the safety of a liquid blend of kava and kratom (FFCT) in healthy adults over six consecutive days of supplementation. Individually, both kava and kratom have been shown to be generally safe when used at moderate dosages over short periods, though concerns about adverse effects, particularly with long-term or high-dosage use, have been noted [5,12,14]. To our knowledge, this is the first published clinical trial demonstrating the safety of a product combining both kava and kratom.

Overall, results from this study indicate that FFCT was generally safe, with only mild to moderate AEs reported, which were all transient in nature. There were no clinically significant trends in laboratory results, including liver and kidney function (P > 0.05). This is a crucial finding considering the historical concerns regarding kava and kratom hepatotoxicity [5,15,16]. These concerns predominantly stem from the use of non-standardized or contaminated products [17]. However, when kratom and kava are used in controlled, standardized dosages, as in this study, the risk of hepatotoxicity appears minimal. Moreover, while kratom may exert dose-dependent effects on both the cardiovascular and respiratory systems, particularly at high doses, the small but statistically significant changes in blood pressure (P < 0.05, in the LD and MD periods) and respiratory rate (P < 0.05, in the HD period) observed during this study were deemed not clinically relevant by the principal investigator [15,18]. All vital sign values, including respiratory rates, remained within normal physiological ranges during this study.

Kratom, and to a lesser extent kava, has been associated with withdrawal symptoms at higher doses [12,14,19]. However, withdrawal symptoms, as measured by COWS and SOWS in this study, remained largely unchanged across all treatment groups, and the total COWS score remained below 5, indicating no withdrawal. There was a small statistically significant increase compared to placebo in total COWS score in the HD period on day 7 (P < 0.05); however, the only COWS symptom score that significantly increased compared to placebo was resting pulse rate. Despite reaching statistical significance, there were no clinically significant changes as per the definition of withdrawal symptom severity based on COWS scoring [11].

Kava use has been found to produce mild adverse effects such as nausea and fatigue, specifically when certain cultivars are used or when consumed as part of an herbal remedy [15]. Similarly, kratom use is associated with nausea, dizziness, and headaches, particularly at higher doses [20]. In this study, adverse events were predominantly mild in nature, with the most common adverse events being nausea, headaches, and fatigue, particularly in the HD period. While the incidence of adverse events increased with higher doses, they remained mild to moderate and resolved before the end of the study. Also, these adverse events were generally considered unpleasant and would therefore be less likely to encourage repeated use or abuse. No serious adverse events were reported. These results are consistent with previous studies evaluating kava and kratom individually [12,15].

A limitation of this study is that it was limited to six consecutive days of use at each dosage level, which may not capture potential adverse effects associated with longer-term use. However, as this is the first-in-human study on a kava and kratom formulation, evaluating shorter-term safety provides a critical foundation for future research investigating longer-term effects. Another limitation is that this study focused exclusively on the safety of the kava and kratom combination and did not include specific measures of tolerability, which would provide further insight into the product's acceptability alongside its safety profile. As tolerability would require longer observation periods to comprehensively assess, future trials should aim to include both longer-term safety and tolerability measures to better understand the product's overall acceptability.

## Conclusions

In conclusion, this study demonstrated that FFCT at the three dosages investigated is safe over six days of supplementation without clinically significant impacts on vital signs, laboratory values, or withdrawal symptoms. The safety profile of FFCT appeared favorable for this short-term use at the investigated dosages. If further research is conducted, it should further explore the subjective effects of kava and kratom in combination.

## Appendices

Table 7 and Table 8 show the chemical chemistry test. 

**Table 7:** Clinical Chemistry Test Shift From Baseline to Day 6 This shift table illustrates changes in clinical chemistry parameters from baseline to day 6. Results are categorized as low, normal, or high based on predefined reference ranges. Each cell in the table represents the number and percentage of the participants who fall into each category at a specific time point. Rows labeled "low," "normal," and "high" indicate the status of the parameter on day 6, while columns under each dosage group indicate the baseline status of the participants in that group. At baseline, n = 39, 40, 40, and 38 for placebo, low-dosage (LD), mid-dosage (MD), and high-dosage (HD) groups, respectively. At day 6, n = 39, 40, 39, and 38 for placebo, low-dosage, mid-dosage, and high-dosage groups, respectively FFCT: Feel Free® Classic Tonic

							FFCT Dosage Levels											
			Placebo Period Baseline				LD Period (15 mL) Baseline				MD Period (15 mL + 15 mL) Baseline				HD Period (30 mL+ 30 mL) Baseline			
Clinical Chemistry Parameter	Study Visit	Status	Low		Normal	High	Low	Normal		High	Low		Normal	High	Low	Normal		High
Albumin (g/L)	Day 6	Low	0 (0%)	0 (0%)		0 (0%)	0 (0%)	0 (0%)	0 (0%)		0 (0%)		0 (0%)	0 (0%)	0 (0%)	0 (0%)		0 (0%)
		Normal	0 (0%)	39 (100%)		0 (0%)	0 (0%)	40 (100%)	0 (0%)		0 (0%)		39 (97.5%)	0 (0%)	0 (0%)	38 (100%)		0 (0%)
		High	0 (0%)	0 (0%)		0 (0%)	0 (0%)	0 (0%)	0 (0%)		0 (0%)		0 (0%)	0 (0%)	0 (0%)	0 (0%)		0 (0%)
Alkaline phosphatase (U/L)	Day 6	Low	0 (0%)	0 (0%)		0 (0%)	0 (0%)	1 (2.5%)	0 (0%)		1 (2.5%)		0 (0%)	0 (0%)	1 (2.6%)	0 (0%)		0 (0%)
		Normal	1 (2.6%)	38 (97.4%)		0 (0%)	0 (0%)	38 (95.0%)	1 (2.5%)		0 (0%)		36 (90.0%)	1 (2.5%)	0 (0%)	37 (97.4%)		0 (0%)
		High	0 (0%)	0 (0%)		0 (0%)	0 (0%)	0 (0%)	0 (0%)		0 (0%)		1 (2.5%)	0 (0%)	0 (0%)	0 (0%)		0 (0%)
Alanine aminotransferase (U/L)	Day 6	Low	0 (0%)	0 (0%)		0 (0%)	0 (0%)	0 (0%)	0 (0%)		0 (0%)		0 (0%)	0 (0%)	0 (0%)	0 (0%)		0 (0%)
		Normal	0 (0%)	36 (92.3%)		1 (2.6%)	0 (0%)	38 (95.0%)	1 (2.5%)		0 (0%)		39 (97.5%)	0 (0%)	0 (0%)	37 (97.4%)		0 (0%)
		High	0 (0%)	1 (2.6%)		1 (2.6%)	0 (0%)	0 (0%)	1 (2.5%)		0 (0%)		0 (0%)	0 (0%)	0 (0%)	0 (0%)		1 (2.6%)
Aspartate aminotransferase (U/L)	Day 6	Low	0 (0%)	0 (0%)		0 (0%)	0 (0%)	0 (0%)	0 (0%)		0 (0%)		0 (0%)	0 (0%)	0 (0%)	0 (0%)		0 (0%)
		Normal	0 (0%)	36 (92.3%)		2 (5.1%)	0 (0%)	39 (97.5%)	1 (2.5%)		0 (0%)		38 (95.0%)	1 (2.5%)	0 (0%)	37 (97.4%)		0 (0%)
		High	0 (0%)	1 (2.6%)		0 (0%)	0 (0%)	0 (0%)	0 (0%)		0 (0%)		0 (0%)	0 (0%)	0 (0%)	0 (0%)		1 (2.6%)
Total bilirubin (umol/L)	Day 6	Low	0 (0%)	0 (0%)		0 (0%)	0 (0%)	0 (0%)	0 (0%)		0 (0%)		1 (2.5%)	0 (0%)	0 (0%)	0 (0%)		0 (0%)
		Normal	0 (0%)	37 (94.9%)		2 (5.1%)	0 (0%)	38 (95.0%)	0 (0%)		0 (0%)		37 (92.5%)	1 (2.5%)	0 (0%)	37 (97.4%)		0 (0%)
		High	0 (0%)	0 (0%)		0 (0%)	0 (0%)	1 (2.5%)	1 (2.5%)		0 (0%)		0 (0%)	0 (0%)	0 (0%)	1 (2.6%)		0 (0%)
Chloride (mmol/L)	Day 6	Low	0 (0%)	0 (0%)		0 (0%)	0 (0%)	0 (0%)	0 (0%)		0 (0%)		1 (2.5%)	0 (0%)	0 (0%)	0 (0%)		0 (0%)
		Normal	0 (0%)	39 (100%)		0 (0%)	0 (0%)	40 (100%)	0 (0%)		0 (0%)		38 (95.0%)	0 (0%)	0 (0%)	38 (100%)		0 (0%)
		High	0 (0%)	0 (0%)		0 (0%)	0 (0%)	0 (0%)	0 (0%)		0 (0%)		0 (0%)	0 (0%)	0 (0%)	0 (0%)		0 (0%)
Creatinine (umol/L)	Day 6	Low	0 (0%)	0 (0%)		0 (0%)	0 (0%)	0 (0%)	0 (0%)		0 (0%)		1 (2.5%)	0 (0%)	0 (0%)	0 (0%)		0 (0%)
		Normal	0 (0%)	39 (100%)		0 (0%)	0 (0%)	38 (95.0%)	1 (2.5%)		0 (0%)		37 (92.5%)	1 (2.5%)	0 (0%)	37 (97.4%)		0 (0%)
		High	0 (0%)	0 (0%)		0 (0%)	0 (0%)	1 (2.5%)	0 (0%)			0 (0%)	0 (0%)	0 (0%)	0 (0%)	1 (2.6%)		0 (0%)
Glomerular filtration rate, estimated (mL/minute/1.73 m^2^)	Day 6	Low	0 (0%)	0 (0%)		0 (0%)	0 (0%)	0 (0%)	0 (0%)			0 (0%)	0 (0%)	0 (0%)	0 (0%)	0 (0%)		0 (0%)
		Normal	0 (0%)	39 (100%)		0 (0%)	0 (0%)	40 (100%)	0 (0%)			1 (2.5%)	38 (95.0%)	0 (0%)	0 (0%)	38 (100%)		0 (0%)
		High	0 (0%)	0 (0%)		0 (0%)	0 (0%)	0 (0%)	0 (0%)			0 (0%)	0 (0%)	0 (0%)	0 (0%)	0 (0%)		0 (0%)
Gamma-glutamyl transferase (U/L)	Day 6	Low	0 (0%)	0 (0%)		0 (0%)	0 (0%)	0 (0%)	0 (0%)			0 (0%)	0 (0%)	0 (0%)	0 (0%)	0 (0%)		0 (0%)
		Normal	0 (0%)	38 (97.4%)		0 (0%)	0 (0%)	37 (92.5%)	0 (0%)			0 (0%)	38 (95.0%)	0 (0%)	0 (0%)	37 (97.4%)		0 (0%)
		High	0 (0%)	0 (0%)		1 (2.6%)	0 (0%)	0 (0%)	3 (7.5%)			0 (0%)	0 (0%)	1 (2.5%)	0 (0%)	0 (0%)		1 (2.6%)
Glucose fasting (mmol/L)	Day 6	Low	0 (0%)	1 (2.6%)		0 (0%)	0 (0%)	0 (0%)	0 (0%)			0 (0%)	1 (2.5%)	0 (0%)	0 (0%)	0 (0%)	0 (0%)	
		Normal	0 (0%)	37 (94.9%)		0 (0%)	0 (0%)	39 (97.5%)	0 (0%)			0 (0%)	35 (87.5%)	1 (2.5%)	0 (0%)	37 (97.4%)	0 (0%)	
		High	0 (0%)	1 (2.6%)		0 (0%)	0 (0%)	0 (0%)	1 (2.5%)			0 (0%)	0 (0%)	2 (5.0%)	0 (0%)	0 (0%)	1 (2.6%)	
Potassium (mmol/L)	Day 6	Low	0 (0%)	0 (0%)		0 (0%)	0 (0%)	1 (2.5%)	0 (0%)			0 (0%)	0 (0%)	0 (0%)	0 (0%)	0 (0%)	0 (0%)	
		Normal	0 (0%)	39 (100%)		0 (0%)	0 (0%)	39 (97.5%)	0 (0%)			0 (0%)	39 (97.5%)	0 (0%)	1 (2.6%)	36 (94.7%)	1 (2.6%)	
		High	0 (0%)	0 (0%)		0 (0%)	0 (0%)	0 (0%)	0 (0%)			0 (0%)	0 (0%)	0 (0%)	0 (0%)	0 (0%)	0 (0%)	
Protein (g/L)	Day 6	Low	0 (0%)	0 (0%)		0 (0%)	0 (0%)	2 (5.0%)	0 (0%)			0 (0%)	0 (0%)	0 (0%)	0 (0%)	0 (0%)	0 (0%)	
		Normal	0 (0%)	39 (100%)		0 (0%)	0 (0%)	37 (92.5%)	1 (2.5%)			0 (0%)	39 (97.5%)	0 (0%)	1 (2.6%)	37 (97.4%)	0 (0%)	
		High	0 (0%)	0 (0%)		0 (0%)	0 (0%)	0 (0%)	0 (0%)			0 (0%)	0 (0%)	0 (0%)	0 (0%)	0 (0%)	0 (0%)	
Sodium (mmol/L)	Day 6	Low	0 (0%)	3 (7.7%)		0 (0%)	1 (2.5%)	0 (0%)	0 (0%)			0 (0%)	1 (2.5%)	0 (0%)	0 (0%)	5 (13.2%)	0 (0%)	
		Normal	1 (2.6%)	35 (89.7%)		0 (0%)	0 (0%)	39 (97.5%)	0 (0%)			2 (5.0%)	36 (90.0%)	0 (0%)	0 (0%)	33 (86.8%)	0 (0%)	
		High	0 (0%)	0 (0%)		0 (0%)	0 (0%)	0 (0%)	0 (0%)			0 (0%)	0 (0%)	0 (0%)	0 (0%)	0 (0%)	0 (0%)	
Urea (mmol/L)	Day 6	Low	1 (2.6%)	0 (0%)		0 (0%)	1 (2.5%)	2 (5.0%)	0 (0%)			0 (0%)	0 (0%)	0 (0%)	0 (0%)	1 (2.6%)	0 (0%)	
		Normal	0 (0%)	37 (94.9%)		0 (0%)	2 (5.0%)	33 (82.5%)	2 (5.0%)			2 (5.0%)	36 (90.0%)	1 (2.5%)	1 (2.6%)	35 (92.1%)	1 (2.6%)	
		High	0 (0%)	0 (0%)		1 (2.6%)	0 (0%)	0 (0%)	0 (0%)			0 (0%)	0 (0%)	0 (0%)	0 (0%)	0 (0%)	0 (0%)	

**Table 8:** Change From Baseline to Day 6 in Clinical Chemistry Values are mean ± SD. At baseline, n = 39, 40, 40, and 38 for placebo, low-dosage, mid-dosage, and high-dosage groups, respectively. At day 6, n = 39, 40, 39, and 38 for placebo, low-dosage, mid-dosage, and high-dosage groups, respectively †A significant between-group difference compared to placebo (P < 0.05) *Significant within-group change from baseline to day 6 (P < 0.05) FFCT, Feel Free® Classic Tonic; Δ, change from baseline

			FFCT Dosage Levels		
Clinical Chemistry Parameter	Study Visit	Placebo	Low Dosage (15 mL)	Mid-dosage (15 mL + 15 mL)	High Dosage (30 mL + 30 mL)
Albumin (g/L)	Baseline	44.8 ± 2.47	44.7 ± 2.55	44.5 ± 1.91	44.3 ± 1.95
	Day 6	44.4 ± 2.51	44.5 ± 2.85	43.6 ± 2.22	44.4 ± 2.26
	Δ	-0.4 ± 2.14	-0.2 ± 2.22	-1.0 ± 2.15*	0.1 ± 2.07
Alkaline phosphatase (U/L)	Baseline	67.8 ± 17.58	72.1 ± 21.30	71.4 ± 22.08	70.0 ± 20.85
	Day 6	68.7 ± 19.54	72.2 ± 22.32	72.7 ± 23.35	74.3 ± 22.07
	Δ	0.9 ± 4.53	0.1 ± 5.93	1.1 ± 4.35	4.3 ± 5.09
Alanine aminotransferase (U/L)	Baseline	19.0 ± 12.84	20.7 ± 12.37	18.9 ± 6.82	19.4 ± 7.84
	Day 6	20.1 ± 12.58	18.5 ± 8.26	18.7 ± 7.68	18.1 ± 8.73
	Δ	1.2 ± 11.48	-2.2 ± 7.50	-0.3 ± 3.93	-1.3 ± 3.99†*
Aspartate aminotransferase (U/L)	Baseline	20.9 ± 7.97	21.2 ± 8.22	20.2 ± 5.62	20.6 ± 8.88
	Day 6	23.1 ± 22.72	19.7 ± 5.11	19.6 ± 4.84	20.0 ± 5.65
	Δ	2.2 ± 22.86	-1.5 ± 6.24	-0.5 ± 4.63	-0.6 ± 5.11†*
Total bilirubin (umol/L)	Baseline	9.2 ± 6.23	8.7 ± 5.02	8.4 ± 4.44	8.3 ± 4.43
	Day 6	8.4 ± 5.13	8.6 ± 5.22	7.5 ± 3.71	7.1 ± 5.00
	Δ	-0.8 ± 4.50	-0.1 ± 3.45	-1.1 ± 3.42	-1.2 ± 3.77*
Chloride (mmol/L)	Baseline	101.9 ± 2.50	101.9 ± 2.23	101.9 ± 1.73	102.9 ± 2.03
	Day 6	102.1 ± 1.92	102.7 ± 2.01	101.2 ± 2.62	100.4 ± 2.16
	Δ	0.2 ± 2.33	0.8 ± 2.53	-0.6 ± 3.09	-2.4 ± 2.57†*
Creatinine (umol/L)	Baseline	78.1 ± 15.31	78.4 ± 15.35	81.7 ± 15.75	80.5 ± 12.74
	Day 6	78.0 ± 16.33	77.7 ± 14.31	81.3 ± 14.37	84.9 ± 14.80
	Δ	-0.1 ± 4.83	-0.7 ± 4.79	-0.4 ± 8.46	4.4 ± 5.07†*
Glomerular filtration rate, estimated (mL/minute/1.73 m^2^)	Baseline	94.7 ± 15.35	95.2 ± 14.19	92.9 ± 14.48	94.3 ± 11.79
	Day 6	94.5 ± 15.14	96.0 ± 13.78	93.9 ± 13.55	89.3 ± 13.77
	Δ	-0.2 ± 5.22	0.8 ± 5.76	1.2 ± 9.29	-4.9 ± 6.04†*
Gamma-glutamyl transferase (U/L)	Baseline	17.4 ± 13.01	19.4 ± 18.27	17.5 ± 12.70	17.6 ± 11.34
	Day 6	17.7 ± 14.16	18.9 ± 14.99	19.9 ± 16.12	20.8 ± 13.19
	Δ	0.4 ± 2.50	-0.6 ± 5.60	2.5 ± 4.28†*	3.2 ± 4.83†*
Glucose fasting (mmol/L)	Baseline	4.89 ± 0.447	4.81 ± 0.497	5.07 ± 0.745	4.96 ± 0.442
	Day 6	4.95 ± 0.550	4.87 ± 0.473	4.91 ± 0.636	4.89 ± 0.510
	Δ	0.06 ± 0.532	0.07 ± 0.531	-0.16 ± 0.545	-0.08 ± 0.389
Potassium (mmol/L)	Baseline	4.52 ± 0.444	4.47 ± 0.353	4.53 ± 0.468	4.57 ± 0.457
	Day 6	4.48 ± 0.311	4.57 ± 0.398	4.59 ± 0.327	4.57 ± 0.387
	Δ	-0.04 ± 0.324	0.11 ± 0.346	0.07 ± 0.403	0.00 ± 0.349
Protein (g/L)	Baseline	69.9 ± 4.43	70.1 ± 5.28	69.4 ± 4.23	69.3 ± 4.16
	Day 6	68.3 ± 3.44	68.7 ± 4.54	69.4 ± 3.95	70.4 ± 4.19
	Δ	-1.6 ± 3.23*	-1.5 ± 3.59*	0.2 ± 3.15†	1.1 ± 3.18†*
Sodium (mmol/L)	Baseline	140.0 ± 2.12	140.2 ± 1.89	139.0 ± 1.82	138.8 ± 1.72
	Day 6	138.9 ± 2.23	139.4 ± 2.12	138.5 ± 2.85	137.8 ± 2.02
	Δ	-1.1 ± 2.08*	-0.8 ± 2.34*	-0.4 ± 3.19†	-1.1 ± 2.12*
Urea (mmol/L)	Baseline	4.88 ± 1.483	4.90 ± 1.835	4.86 ± 1.616	4.59 ± 1.523
	Day 6	4.82 ± 1.530	4.83 ± 1.517	4.73 ± 0.975	4.36 ± 0.909
	Δ	-0.06 ± 1.131	-0.06 ± 1.235	-0.12 ± 1.289	-0.23 ± 1.464

Table 9 and Table 10 show the hematology test.

**Table 9:** Hematology Test Shift From Baseline to Day 6 This shift table illustrates changes in clinical chemistry parameters from baseline to day 6. Results are categorized as low, normal, or high based on predefined reference ranges. Each cell in the table represents the number and percentage of the participants who fall into each category at a specific time point. Rows labeled "low," "normal," and "high" indicate the status of the parameter at day 6, while columns under each dosage group indicate the baseline status of the participants in that group. At baseline, n = 39, 40, 40, and 38 for placebo, low-dosage (LD), mid-dosage (LD), and high-dosage (HD) groups, respectively. At day 6, n = 39, 40, 39, and 38 for placebo, low-dosage, mid-dosage, and high-dosage groups, respectively FFCT: Feel Free® Classic Tonic

						FFCT Dosage Levels								
			Placebo Period Baseline			LD Period (15 mL) Baseline			MD Period (15 mL + 15 mL) Baseline			HD Period (30 mL + 30 mL) Baseline		
Hematology Parameter	Study Visit	Status	Low	Normal	High	Low	Normal	High	Low	Normal	High	Low	Normal	High
Basophils (10^9^/L)	Day 6	Low	0 (0%)	0 (0%)	0 (0%)	0 (0%)	0 (0%)	0 (0%)	0 (0%)	0 (0%)	0 (0%)	0 (0%)	0 (0%)	0 (0%)
		Normal	0 (0%)	39 (100%)	0 (0%)	0 (0%)	40 (100%)	0 (0%)	0 (0%)	39 (97.5%)	0 (0%)	0 (0%)	37 (97.4%)	0 (0%)
		High	0 (0%)	0 (0%)	0 (0%)	0 (0%)	0 (0%)	0 (0%)	0 (0%)	0 (0%)	0 (0%)	0 (0%)	1 (2.6%)	0 (0%)
Eosinophils (10^9^/L)	Day 6	Low	0 (0%)	0 (0%)	0 (0%)	0 (0%)	0 (0%)	0 (0%)	0 (0%)	0 (0%)	0 (0%)	0 (0%)	0 (0%)	0 (0%)
		Normal	0 (0%)	37 (94.9%)	0 (0%)	0 (0%)	38 (95.0%)	0 (0%)	0 (0%)	38 (95.0%)	0 (0%)	0 (0%)	35 (92.1%)	1 (2.6%)
		High	0 (0%)	1 (2.6%)	1 (2.6%)	0 (0%)	1 (2.5%)	1 (2.5%)	0 (0%)	1 (2.5%)	0 (0%)	0 (0%)	2 (5.3%)	0 (0%)
Hematocrit (L/L)	Day 6	Low	0 (0%)	1 (2.6%)	0 (0%)	0 (0%)	0 (0%)	0 (0%)	0 (0%)	0 (0%)	0 (0%)	1 (2.6%)	0 (0%)	0 (0%)
		Normal	0 (0%)	37 (94.9%)	0 (0%)	0 (0%)	39 (97.5%)	0 (0%)	0 (0%)	38 (95.0%)	1 (2.5%)	0 (0%)	36 (94.7%)	0 (0%)
		High	0 (0%)	1 (2.6%)	0 (0%)	0 (0%)	1 (2.5%)	0 (0%)	0 (0%)	0 (0%)	0 (0%)	0 (0%)	1 (2.6%)	0 (0%)
Hemoglobin (HGB) (g/L)	Day 6	Low	0 (0%)	2 (5.1%)	0 (0%)	0 (0%)	0 (0%)	0 (0%)	0 (0%)	1 (2.5%)	0 (0%)	1 (2.6%)	0 (0%)	0 (0%)
		Normal	0 (0%)	37 (94.9%)	0 (0%)	0 (0%)	39 (97.5%)	0 (0%)	0 (0%)	37 (92.5%)	0 (0%)	0 (0%)	35 (92.1%)	0 (0%)
		High	0 (0%)	0 (0%)	0 (0%)	0 (0%)	0 (0%)	1 (2.5%)	0 (0%)	0 (0%)	1 (2.5%)	0 (0%)	2 (5.3%)	0 (0%)
Lymphocytes (10^9^/L)	Day 6	Low	1 (2.6%)	1 (2.6%)	0 (0%)	1 (2.5%)	0 (0%)	0 (0%)	1 (2.5%)	1 (2.5%)	0 (0%)	0 (0%)	3 (7.9%)	0 (0%)
		Normal	2 (5.1%)	34 (87.2%)	1 (2.6%)	2 (5.0%)	36 (90.0%)	1 (2.5%)	2 (5.0%)	34 (85.0%)	1 (2.5%)	0 (0%)	34 (89.5%)	1 (2.6%)
		High	0 (0%)	0 (0%)	0 (0%)	0 (0%)	0 (0%)	0 (0%)	0 (0%)	0 (0%)	0 (0%)	0 (0%)	0 (0%)	0 (0%)
Mean corpuscular hemoglobin (pg)	Day 6	Low	0 (0%)	0 (0%)	0 (0%)	0 (0%)	0 (0%)	0 (0%)	0 (0%)	0 (0%)	0 (0%)	0 (0%)	0 (0%)	0 (0%)
		Normal	0 (0%)	39 (100%)	0 (0%)	0 (0%)	39 (97.5%)	0 (0%)	0 (0%)	38 (95.0%)	1 (2.5%)	0 (0%)	37 (97.4%)	0 (0%)
		High	0 (0%)	0 (0%)	0 (0%)	0 (0%)	1 (2.5%)	0 (0%)	0 (0%)	0 (0%)	0 (0%)	0 (0%)	1 (2.6%)	0 (0%)
Mean corpuscular HGB concentration (g/L)	Day 6	Low	0 (0%)	0 (0%)	0 (0%)	0 (0%)	1 (2.5%)	0 (0%)	0 (0%)	0 (0%)	0 (0%)	0 (0%)	0 (0%)	0 (0%)
		Normal	0 (0%)	37 (94.9%)	0 (0%)	0 (0%)	33 (82.5%)	2 (5.0%)	0 (0%)	34 (85.0%)	3 (7.5%)	0 (0%)	33 (86.8%)	1 (2.6%)
		High	0 (0%)	1 (2.6%)	1 (2.6%)	0 (0%)	3 (7.5%)	1 (2.5%)	0 (0%)	2 (5.0%)	0 (0%)	0 (0%)	3 (7.9%)	1 (2.6%)
Mean corpuscular volume (fL)	Day 6	Low	0 (0%)	0 (0%)	0 (0%)	0 (0%)	0 (0%)	0 (0%)	3 (7.5%)	0 (0%)	0 (0%)	3 (7.9%)	0 (0%)	0 (0%)
		Normal	0 (0%)	39 (100%)	0 (0%)	0 (0%)	39 (97.5%)	0 (0%)	0 (0%)	35 (87.5%)	0 (0%)	0 (0%)	33 (86.8%)	1 (2.6%)
		High	0 (0%)	0 (0%)	0 (0%)	0 (0%)	1 (2.5%)	0 (0%)	0 (0%)	1 (2.5%)	0 (0%)	0 (0%)	0 (0%)	1 (2.6%)
Monocytes (10^9^/L)	Day 6	Low	0 (0%)	0 (0%)	0 (0%)	0 (0%)	0 (0%)	0 (0%)	0 (0%)	0 (0%)	0 (0%)	0 (0%)	1 (2.6%)	0 (0%)
		Normal	0 (0%)	37 (94.9%)	1 (2.6%)	0 (0%)	39 (97.5%)	1 (2.5%)	0 (0%)	37 (92.5%)	2 (5.0%)	0 (0%)	37 (97.4%)	0 (0%)
		High	0 (0%)	1 (2.6%)	0 (0%)	0 (0%)	0 (0%)	0 (0%)	0 (0%)	0 (0%)	0 (0%)	0 (0%)	0 (0%)	0 (0%)
Mean platelet volume (fL)	Day 6	Low	0 (0%)	0 (0%)	0 (0%)	0 (0%)	0 (0%)	0 (0%)	0 (0%)	0 (0%)	0 (0%)	0 (0%)	0 (0%)	0 (0%)
		Normal	0 (0%)	39 (100%)	0 (0%)	0 (0%)	40 (100%)	0 (0%)	0 (0%)	39 (97.5%)	0 (0%)	0 (0%)	38 (100%)	0 (0%)
		High	0 (0%)	0 (0%)	0 (0%)	0 (0%)	0 (0%)	0 (0%)	0 (0%)	0 (0%)	0 (0%)	0 (0%)	0 (0%)	0 (0%)
Neutrophils (10^9^/L)	Day 6	Low	1 (2.6%)	3 (7.7%)	0 (0%)	0 (0%)	1 (2.5%)	0 (0%)	0 (0%)	0 (0%)	0 (0%)	0 (0%)	1 (2.6%)	0 (0%)
		Normal	0 (0%)	32 (82.1%)	0 (0%)	1 (2.5%)	38 (95.0%)	0 (0%)	1 (2.5%)	36 (90.0%)	1 (2.5%)	1 (2.6%)	35 (92.1%)	0 (0%)
		High	0 (0%)	2 (5.1%)	1 (2.6%)	0 (0%)	0 (0%)	0 (0%)	0 (0%)	1 (2.5%)	0 (0%)	0 (0%)	1 (2.6%)	0 (0%)
Platelets (10^9^/L)	Day 6	Low	0 (0%)	0 (0%)	0 (0%)	0 (0%)	2 (5.0%)	0 (0%)	1 (2.5%)	0 (0%)	0 (0%)	1 (2.6%)	1 (2.6%)	0 (0%)
		Normal	0 (0%)	36 (92.3%)	1 (2.6%)	0 (0%)	35 (87.5%)	0 (0%)	0 (0%)	36 (90.0%)	1 (2.5%)	0 (0%)	34 (89.5%)	0 (0%)
		High	0 (0%)	2 (5.1%)	0 (0%)	0 (0%)	2 (5.0%)	1 (2.5%)	0 (0%)	1 (2.5%)	0 (0%)	0 (0%)	2 (5.3%)	0 (0%)
Erythrocytes (10^12^/L)	Day 6	Low	1 (2.6%)	0 (0%)	0 (0%)	0 (0%)	1 (2.5%)	0 (0%)	0 (0%)	1 (2.5%)	0 (0%)	1 (2.6%)	1 (2.6%)	0 (0%)
		Normal	0 (0%)	38 (97.4%)	0 (0%)	1 (2.5%)	38 (95.0%)	0 (0%)	1 (2.5%)	37 (92.5%)	0 (0%)	0 (0%)	36 (94.7%)	0 (0%)
		High	0 (0%)	0 (0%)	0 (0%)	0 (0%)	0 (0%)	0 (0%)	0 (0%)	0 (0%)	0 (0%)	0 (0%)	0 (0%)	0 (0%)
Erythrocyte distribution width	Day 6	Low	1 (2.6%)	1 (2.6%)	0 (0%)	1 (2.5%)	1 (2.5%)	0 (0%)	0 (0%)	0 (0%)	0 (0%)	0 (0%)	3 (7.9%)	0 (0%)
		Normal	2 (5.1%)	33 (84.6%)	1 (2.6%)	0 (0%)	35 (87.5%)	0 (0%)	0 (0%)	37 (92.5%)	0 (0%)	0 (0%)	33 (86.8%)	0 (0%)
		High	0 (0%)	0 (0%)	1 (2.6%)	0 (0%)	0 (0%)	3 (7.5%)	0 (0%)	0 (0%)	2 (5.0%)	0 (0%)	1 (2.6%)	1 (2.6%)
Leukocytes (10^9^/L)	Day 6	Low	0 (0%)	1 (2.6%)	0 (0%)	0 (0%)	1 (2.5%)	0 (0%)	0 (0%)	0 (0%)	0 (0%)	0 (0%)	1 (2.6%)	0 (0%)
		Normal	1 (2.6%)	35 (89.7%)	0 (0%)	2 (5.0%)	37 (92.5%)	0 (0%)	1 (2.5%)	36 (90.0%)	2 (5.0%)	0 (0%)	37 (97.4%)	0 (0%)
		High	0 (0%)	1 (2.6%)	1 (2.6%)	0 (0%)	0 (0%)	0 (0%)	0 (0%)	0 (0%)	0 (0%)	0 (0%)	0 (0%)	0 (0%)

**Table 10:** Change From Baseline to Day 6 in Hematology Values are mean ± SD. At baseline, n = 39, 40, 40, and 38 for placebo, low-dosage (LD), mid-dosage (MD), and high-dosage (HD) groups, respectively. At day 6, n = 39, 40, 39, and 38 for placebo, low-dosage, mid-dosage, and high-dosage groups, respectively †A significant between-group difference compared to placebo (P < 0.05) *Significant within-group change from baseline to day 6 (P < 0.05) FFCT, Feel Free® Classic Tonic; Δ, change from baseline

			FFCT Dosage Levels		
Hematology Parameter	Study Visit	Placebo Period	LD Period (15 mL)	MD Period (15 mL + 15 mL)	HD Period (30 mL + 30 mL)
Basophils (10^9^/L)	Baseline	0.017 ± 0.0274	0.014 ± 0.0253	0.015 ± 0.0242	0.019 ± 0.0261
	Day 6	0.017 ± 0.0251	0.024 ± 0.0282	0.017 ± 0.0255	0.024 ± 0.0336
	Δ	0.001 ± 0.0292	0.010 ± 0.0322	0.001 ± 0.0325	0.005 ± 0.0322
Eosinophils (10^9^/L)	Baseline	0.17 ± 0.204	0.19 ± 0.226	0.16 ± 0.123	0.14 ± 0.111
	Day 6	0.18 ± 0.206	0.19 ± 0.220	0.18 ± 0.141	0.19 ± 0.176
	Δ	0.02 ± 0.081	0.01 ± 0.104	0.03 ± 0.121	0.04 ± 0.141*
Hematocrit (L/L)	Baseline	0.408 ± 0.0373	0.410 ± 0.0378	0.411 ± 0.0410	0.410 ± 0.0378
	Day 6	0.410 ± 0.0403	0.409 ± 0.0386	0.413 ± 0.0372	0.415 ± 0.0387
	Δ	0.002 ± 0.0136	-0.001 ± 0.0163	0.001 ± 0.0152	0.005 ± 0.0148*
Hemoglobin (HGB) (g/L)	Baseline	136.2 ± 13.39	137.0 ± 14.28	137.4 ± 13.63	137.0 ± 13.81
	Day 6	135.8 ± 14.29	136.6 ± 13.75	137.8 ± 13.60	138.9 ± 13.80
	Δ	-0.3 ± 4.11	-0.4 ± 5.20	0.2 ± 3.60	1.9 ± 4.88†*
Lymphocytes (10^9^/L)	Baseline	1.65 ± 0.552	1.71 ± 0.504	1.72 ± 0.547	1.66 ± 0.504
	Day 6	1.63 ± 0.440	1.70 ± 0.393	1.56 ± 0.466	1.50 ± 0.506
	Δ	-0.03 ± 0.317	-0.02 ± 0.299	-0.18 ± 0.331†*	-0.16 ± 0.331*
Mean corpuscular hemoglobin (pg)	Baseline	29.2 ± 1.93	29.1 ± 2.06	29.4 ± 2.09	29.4 ± 2.33
	Day 6	29.1 ± 2.05	29.2 ± 2.07	29.4 ± 2.07	29.5 ± 2.25
	Δ	-0.1 ± 0.51	0.1 ± 0.61	0.0 ± 0.58	0.1 ± 0.74
Mean corpuscular HGB concentration (g/L)	Baseline	333.2 ± 6.37	334.0 ± 9.04	334.4 ± 6.32	334.4 ± 7.64
	Day 6	331.7 ± 6.79	333.8 ± 7.70	334.0 ± 7.41	335.0 ± 7.25
	Δ	-1.5 ± 4.15	-0.2 ± 6.83	-0.5 ± 6.36	0.5 ± 6.56
Mean corpuscular volume (fL)	Baseline	87.5 ± 5.07	87.2 ± 4.87	87.9 ± 5.37	87.8 ± 5.79
	Day 6	87.7 ± 5.13	87.4 ± 4.84	88.0 ± 5.26	87.9 ± 5.60
	Δ	0.2 ± 0.67	0.2 ± 0.88	-0.1 ± 0.84	0.2 ± 0.92
Monocytes (10^9^/L)	Baseline	0.48 ± 0.152	0.46 ± 0.157	0.49 ± 0.195	0.45 ± 0.139
	Day 6	0.46 ± 0.158	0.46 ± 0.143	0.44 ± 0.150	0.42 ± 0.128
	Δ	-0.02 ± 0.123	0.00 ± 0.109	-0.04 ± 0.137*	-0.03 ± 0.109
Mean platelet volume (fL)	Baseline	9.34 ± 1.103	9.30 ± 0.997	9.07 ± 1.068	9.21 ± 1.030
	Day 6	9.35 ± 1.074	9.32 ± 1.067	9.30 ± 1.124	9.29 ± 1.024
	Δ	0.01 ± 0.310	0.02 ± 0.301	0.23 ± 0.410†*	0.08 ± 0.285
Neutrophils (10^9^/L)	Baseline	3.51 ± 1.975	3.17 ± 1.225	3.24 ± 1.428	3.08 ± 0.967
	Day 6	3.21 ± 1.514	3.21 ± 1.177	3.23 ± 1.279	3.17 ± 1.263
	Δ	-0.30 ± 1.369	0.04 ± 0.792	0.03 ± 0.991	0.09 ± 1.213
Platelets (10^9^/L)	Baseline	253.0 ± 66.50	253.3 ± 59.75	255.1 ± 59.94	257.1 ± 56.15
	Day 6	250.6 ± 65.48†*	262.4 ± 65.59	253.3 ± 57.18	255.9 ± 63.30
	Δ	-2.3 ± 20.25	9.2 ± 26.45	0.8 ± 15.85	-1.2 ± 24.46
Erythrocytes (10^12^/L)	Baseline	4.68 ± 0.457	4.70 ± 0.473	4.70 ± 0.538	4.69 ± 0.543
	Day 6	4.68 ± 0.491	4.70 ± 0.468	4.71 ± 0.496	4.74 ± 0.531
	Δ	-0.00 ± 0.152*	-0.01 ± 0.182*	0.00 ± 0.161*	0.04 ± 0.164*
Erythrocyte distribution width	Baseline	13.91 ± 1.497	13.91 ± 1.634	14.07 ± 1.345	13.93 ± 1.091
	Day 6	13.89 ± 1.494	13.88 ± 1.638	14.02 ± 1.281	13.93 ± 1.218
	Δ	-0.02 ± 0.244	-0.03 ± 0.234	-0.02 ± 0.243	0.00 ± 0.307
Leukocytes (10^9^/L)	Baseline	5.95 ± 2.298	5.67 ± 1.672	5.73 ± 1.744	5.45 ± 1.326
	Day 6	5.61 ± 1.829	5.69 ± 1.581	5.55 ± 1.615	5.41 ± 1.402
	Δ	-0.33 ± 1.432	0.02 ± 0.945	-0.14 ± 1.012	-0.04 ± 1.128

Table 11 and Table 12 show the summary of the sub-group analysis for clinical chemistry and hematology.

**Table 11:** Change From Baseline to Day 6 in Clinical Chemistry (Sub-group Analysis) Values are mean ± SD. At baseline and day 6, n = 25, 25, 25, and 25 for high-dosage (HD), mid-dosage (MD), low-dosage (LD), and placebo groups, respectively †A significant between-group difference compared to placebo (P < 0.05) *Significant within-group change from baseline to day 6 (P < 0.05) FFCT, Feel Free® Classic Tonic; Δ, change from baseline

			FFCT Dosage Levels		
Clinical Chemistry Parameter	Study Visit	Placebo Period	LD Period (15 mL)	MD Period (15 mL + 15 mL)	HD Period (30 mL + 30 mL)
Albumin (g/L)	Baseline	44.6 ± 2.69	44.1 ± 2.44	44.2 ± 2.05	44.1 ± 2.18
	Day 6	43.8 ± 2.59	43.9 ± 3.20	43.4 ± 2.29	44.4 ± 2.53
	Δ	-0.8 ± 2.13	-0.2 ± 2.36	-0.9 ± 2.22	0.3 ± 2.12
Alkaline phosphatase (U/L)	Baseline	69.9 ± 16.92	73.3 ± 20.15	69.4 ± 16.58	69.2 ± 18.39
	Day 6	70.2 ± 19.03	73.5 ± 21.93	70.8 ± 18.36	73.6 ± 19.86
	Δ	0.4 ± 4.08	0.2 ± 6.33	1.4 ± 4.59	4.3 ± 5.81†*
Alanine aminotransferase (U/L)	Baseline	16.9 ± 6.55	18.7 ± 6.87	17.6 ± 5.12	18.8 ± 6.09
	Day 6	19.2 ± 12.34	16.6 ± 5.45	17.5 ± 6.44	16.8 ± 6.60
	Δ	2.4 ± 11.55	-2.0 ± 5.01	-0.1 ± 4.33	-2.1 ± 4.40†*
Aspartate aminotransferase (U/L)	Baseline	19.5 ± 4.93	20.2 ± 3.47	19.1 ± 4.16	19.3 ± 4.26
	Day 6	24.6 ± 27.88	19.0 ± 3.90	19.2 ± 4.50	19.1 ± 4.58
	Δ	5.1 ± 27.72	-1.1 ± 3.31	0.1 ± 3.16	-0.2 ± 3.25
Total bilirubin (umol/L)	Baseline	8.7 ± 6.44	8.4 ± 5.09	8.3 ± 3.78	8.1 ± 4.38
	Day 6	7.8 ± 5.03	8.1 ± 5.35	7.7 ± 4.14	7.0 ± 3.94
	Δ	-0.9 ± 5.08	-0.2 ± 3.50	-0.6 ± 3.34	-1.1 ± 2.73
Chloride (mmol/L)	Baseline	101.6 ± 2.66	102.1 ± 1.80	101.8 ± 1.95	103.0 ± 1.84
	Day 6	102.1 ± 1.82	102.4 ± 2.12	101.5 ± 1.76	100.7 ± 2.10
	Δ	0.5 ± 2.31	0.4 ± 2.29	-0.3 ± 2.25	-2.3 ± 2.65†*
Creatinine (umol/L)	Baseline	77.6 ± 15.36	77.1 ± 14.21	79.0 ± 13.05	78.3 ± 11.98
	Day 6	76.9 ± 15.75	76.5 ± 14.19	79.7 ± 13.55	82.4 ± 13.88
	Δ	-0.7 ± 4.88	-0.6 ± 4.31	0.6 ± 5.48	4.1 ± 4.45†*
Glomerular filtration rate, estimated (mL/minute/1.73 m^2^)	Baseline	92.4 ± 15.44	93.2 ± 13.17	93.7 ± 13.33	94.3 ± 12.30
	Day 6	92.7 ± 14.61	93.9 ± 13.74	93.5 ± 14.22	89.8 ± 14.37
	Δ	0.3 ± 5.17	0.7 ± 5.53	-0.2 ± 6.77	-4.4 ± 5.67†*
Gamma-glutamyl transferase (U/L)	Baseline	16.2 ± 6.78	17.6 ± 16.51	14.2 ± 5.59	14.6 ± 5.45
	Day 6	15.9 ± 6.52	16.4 ± 10.86	16.0 ± 8.02	17.4 ± 8.35
	Δ	-0.3 ± 1.35	-1.2 ± 6.32	1.9 ± 3.09†*	2.8 ± 4.97†*
Glucose fasting (mmol/L)	Baseline	4.87 ± 0.467	4.86 ± 0.550	5.14 ± 0.910	5.02 ± 0.493
	Day 6	5.01 ± 0.639	4.87 ± 0.517	4.89 ± 0.735	4.87 ± 0.524
	Δ	0.14 ± 0.624	0.00 ± 0.598	-0.24 ± 0.596*	-0.15 ± 0.355†*
Potassium (mmol/L)	Baseline	4.51 ± 0.420	4.42 ± 0.359	4.57 ± 0.450	4.56 ± 0.443
	Day 6	4.50 ± 0.323	4.59 ± 0.402	4.57 ± 0.322	4.57 ± 0.399
	Δ	-0.02 ± 0.279	0.17 ± 0.363*	0.00 ± 0.396	0.01 ± 0.361
Protein (g/L)	Baseline	70.4 ± 4.86	70.0 ± 5.05	68.6 ± 3.88	68.7 ± 4.34
	Day 6	68.6 ± 3.58	69.1 ± 4.97	69.2 ± 4.25	70.2 ± 4.65
	Δ	-1.8 ± 3.70*	-0.9 ± 3.34	0.6 ± 3.12†	1.5 ± 3.33†*
Sodium (mmol/L)	Baseline	140.3 ± 2.34	140.4 ± 1.91	138.9 ± 2.07	138.8 ± 1.67
	Day 6	139.1 ± 2.25	139.4 ± 2.38	138.9 ± 1.80	138.0 ± 1.87
	Δ	-1.2 ± 1.92*	-1.0 ± 2.53	0.0 ± 1.79†	-0.8 ± 2.09
Urea (mmol/L)	Baseline	4.77 ± 1.232	4.77 ± 1.562	4.86 ± 1.526	4.69 ± 1.678
	Day 6	4.76 ± 1.124	4.65 ± 1.477	4.86 ± 1.126	4.38 ± 0.907
	Δ	-0.01 ± 1.188	-0.12 ± 1.107	0.00 ± 1.069	-0.31 ± 1.669

**Table 12:** Change From Baseline to Day 6 in Hematology (Sub-group Analysis) Values are mean ± SD. At baseline and day 6, n = 25, 25, 25, and 25 for high-dosage (HD), mid-dosage (MD), low-dosage (LD), and placebo groups, respectively †A significant between-group difference compared to placebo (P < 0.05) *Significant within-group change from baseline to day 6 (P < 0.05) FFCT, Feel Free® Classic Tonic; Δ, change from baseline

			FFCT Dosage Levels		
Hematology Parameter	Study Visit	Placebo Period	LD Period (15 mL)	MD Period (15 mL + 15 mL)	HD Period (30 mL + 30 mL)
Basophils (10^9^/L)	Baseline	0.018 ± 0.0254	0.016 ± 0.0277	0.012 ± 0.0230	0.019 ± 0.0262
	Day 6	0.021 ± 0.0266	0.027 ± 0.0278	0.020 ± 0.0262	0.023 ± 0.0276
	Δ	0.003 ± 0.0273	0.010 ± 0.0353	0.008 ± 0.0293	0.004 ± 0.0299
Eosinophils (10^9^/L)	Baseline	0.16 ± 0.123	0.16 ± 0.111	0.16 ± 0.112	0.16 ± 0.119
	Day 6	0.17 ± 0.124	0.20 ± 0.158	0.21 ± 0.148	0.23 ± 0.186
	Δ	0.02 ± 0.090	0.04 ± 0.111	0.05 ± 0.136*	0.07 ± 0.146*
Hematocrit (L/L)	Baseline	0.405 ± 0.0349	0.404 ± 0.0369	0.406 ± 0.0411	0.407 ± 0.0373
	Day 6	0.407 ± 0.0385	0.404 ± 0.0382	0.409 ± 0.0379	0.415 ± 0.0407
	Δ	0.002 ± 0.0122	0.000 ± 0.0173	0.003 ± 0.0154	0.008 ± 0.0162*
Hemoglobin (HBG) (g/L)	Baseline	134.6 ± 12.75	134.3 ± 13.35	136.2 ± 14.19	136.1 ± 14.23
	Day 6	134.5 ± 13.73	134.4 ± 13.47	136.6 ± 14.45	138.8 ± 14.71
	Δ	-0.1 ± 4.07	0.1 ± 5.19	0.4 ± 3.86	2.7 ± 5.61*
Lymphocytes (10^9^/L)	Baseline	1.62 ± 0.611	1.65 ± 0.539	1.76 ± 0.466	1.66 ± 0.433
	Day 6	1.61 ± 0.484	1.63 ± 0.433	1.57 ± 0.449	1.56 ± 0.441
	Δ	-0.01 ± 0.347	-0.02 ± 0.302	-0.18 ± 0.298*	-0.11 ± 0.337
Mean corpuscular hemoglobin (pg)	Baseline	29.0 ± 2.03	28.8 ± 2.19	29.4 ± 2.10	29.2 ± 2.33
	Day 6	29.0 ± 2.15	29.1 ± 2.22	29.3 ± 1.97	29.4 ± 2.20
	Δ	-0.1 ± 0.49	0.2 ± 0.60	-0.1 ± 0.57	0.2 ± 0.80
Mean corpuscular HGB concentration (g/L)	Baseline	332.3 ± 5.93	332.2 ± 8.12	335.4 ± 6.84	334.7 ± 8.11
	Day 6	330.4 ± 6.30	332.3 ± 7.55	334.4 ± 8.09	335.1 ± 8.11
	Δ	-1.9 ± 4.30*	0.1 ± 6.50	-1.0 ± 6.46	0.4 ± 6.85
Mean corpuscular volume (fL)	Baseline	87.3 ± 5.56	87.0 ± 5.43	87.5 ± 5.32	87.3 ± 5.58
	Day 6	87.6 ± 5.58	87.4 ± 5.31	87.5 ± 5.16	87.5 ± 5.54
	Δ	0.2 ± 0.66	0.3 ± 0.85†	0.0 ± 0.65	0.2 ± 0.78
Monocytes (10^9^/L)	Baseline	0.48 ± 0.149	0.43 ± 0.128	0.46 ± 0.122	0.47 ± 0.131
	Day 6	0.45 ± 0.156	0.45 ± 0.119	0.45 ± 0.139	0.45 ± 0.116
	Δ	-0.03 ± 0.131	0.02 ± 0.118	-0.01 ± 0.124	-0.02 ± 0.105
Mean platelet volume (fL)	Baseline	9.34 ± 1.089	9.26 ± 1.004	9.17 ± 1.051	9.42 ± 1.076
	Day 6	9.29 ± 1.003	9.26 ± 1.075	9.48 ± 1.080	9.49 ± 1.067
	Δ	-0.04 ± 0.327	0.01 ± 0.290	0.31 ± 0.363†*	0.06 ± 0.291
Neutrophils (10^9^/L)	Baseline	3.40 ± 1.202	3.05 ± 1.168	3.05 ± 0.958	3.27 ± 1.055
	Day 6	3.14 ± 1.328	3.18 ± 1.135	3.18 ± 1.152	3.10 ± 1.075
	Δ	-0.27 ± 1.046	0.13 ± 0.841	0.13 ± 0.821	-0.17 ± 0.783
Platelets (10^9^/L)	Baseline	254.0 ± 71.63	258.1 ± 64.18	247.6 ± 62.60	251.6 ± 61.55
	Day 6	253.1 ± 71.41	265.3 ± 68.43	247.1 ± 62.82	252.2 ± 72.71
	Δ	-0.8 ± 20.01	7.2 ± 25.23	-0.5 ± 15.23	0.5 ± 26.15
Erythrocytes (10^12^/L)	Baseline	4.66 ± 0.496	4.65 ± 0.522	4.66 ± 0.542	4.68 ± 0.544
	Day 6	4.66 ± 0.532	4.65 ± 0.500	4.68 ± 0.490	4.76 ± 0.553
	Δ	0.01 ± 0.141	0.00 ± 0.187	0.02 ± 0.158	0.07 ± 0.177
Erythrocyte distribution width	Baseline	13.96 ± 1.550	13.95 ± 1.658	14.18 ± 1.543	14.03 ± 1.240
	Day 6	13.95 ± 1.579	13.96 ± 1.725	14.20 ± 1.456	14.02 ± 1.401
	Δ	0.00 ± 0.249	0.01 ± 0.231	0.02 ± 0.246	-0.01 ± 0.326
Leukocytes (10^9^/L)	Baseline	5.79 ± 1.570	5.43 ± 1.582	5.56 ± 1.250	5.68 ± 1.331
	Day 6	5.50 ± 1.582	5.57 ± 1.576	5.54 ± 1.485	5.46 ± 1.362
	Δ	-0.29 ± 1.003	0.14 ± 0.969	-0.02 ± 0.934	-0.23 ± 0.862

Table 13 and Table 14 show the summary of COWS and SOWS scores.

**Table 13:** Change From Baseline to Day 6 in COWS Values are mean ± SD. At baseline, n = 39, 40, 40, and 38 for placebo, low-dosage (LD), mid-dosage (MD), and high-dosage (HD) groups, respectively. At day 7 and day 8, n = 39, 39, 40, and 38 for placebo, low-dosage, mid-dosage, and high-dosage groups, respectively (exceptions: n = 38) ^a^n = 38 †A significant between-group difference compared to placebo (P < 0.05) *Significant within-group change from baseline to day 6 (P < 0.05) FFCT, Feel Free® Classic Tonic; GI, gastrointestinal; Δ, change from baseline; COWS, Clinical Opiate Withdrawal Scale

			FFCT Dosage Levels			
COWS Score	Study Visit	Placebo Period	LD Period (15 mL)	MD Period (15 mL + 15 mL)	HD Period (30 mL + 30 mL)	
						Resting pulse rate	Baseline	0.0 ± 0.16	0.0 ± 0.00	0.2 ± 0.36	0.1 ± 0.23	
Day 7	0.1 ± 0.22	0.1 ± 0.22	0.2 ± 0.47	0.3 ± 0.46		
Δ	0.0 ± 0.28	0.1 ± 0.22	0.1 ± 0.42	0.2 ± 0.49†*		
Day 9	0.1 ± 0.22	0.0 ± 0.00	0.1 ± 0.27	0.2 ± 0.39		
Δ	0.0 ± 0.16	0.0 ± 0.00	-0.1 ± 0.35	0.1 ± 0.41		
Sweating	Baseline	0.0 ± 0.00	0.0 ± 0.00	0.0 ± 0.00	0.0 ± 0.00	
	Day 7	0.0 ± 0.00	0.0 ± 0.00^a^	0.0 ± 0.00	0.0 ± 0.00	
	Δ	0.0 ± 0.00	0.0 ± 0.00	0.0 ± 0.00	0.0 ± 0.00	
	Day 9	0.0 ± 0.00	0.0 ± 0.00^a^	0.0 ± 0.00	0.0 ± 0.00	
	Δ	0.0 ± 0.00	0.0 ± 0.00	0.0 ± 0.00	0.0 ± 0.00	
Restlessness	Baseline	0.0 ± 0.00	0.0 ± 0.00	0.0 ± 0.00	0.0 ± 0.00	
	Day 7	0.0 ± 0.00	0.0 ± 0.00	0.0 ± 0.00	0.0 ± 0.16	
	Δ	0.0 ± 0.00	0.0 ± 0.00	0.0 ± 0.00	0.0 ± 0.16	
	Day 9	0.0 ± 0.00	0.0 ± 0.00	0.0 ± 0.00	0.0 ± 0.00	
	Δ	0.0 ± 0.00	0.0 ± 0.00	0.0 ± 0.00	0.0 ± 0.00	
Pupil size	Baseline	0.0 ± 0.00	0.0 ± 0.00	0.0 ± 0.00	0.0 ± 0.00	
	Day 7	0.0 ± 0.00	0.0 ± 0.00	0.0 ± 0.00	0.0 ± 0.00	
	Δ	0.0 ± 0.00	0.0 ± 0.00	0.0 ± 0.00	0.0 ± 0.00	
	Day 9	0.0 ± 0.00	0.0 ± 0.00	0.0 ± 0.00	0.0 ± 0.00	
	Δ	0.0 ± 0.00	0.0 ± 0.00	0.0 ± 0.00	0.0 ± 0.00	
Bone or joint aches	Baseline	0.0 ± 0.00	0.0 ± 0.00	0.0 ± 0.00	0.0 ± 0.00	
	Day 7	0.0 ± 0.00	0.0 ± 0.16	0.0 ± 0.00	0.0 ± 0.23	
	Δ	0.0 ± 0.00	0.0 ± 0.16	0.0 ± 0.00	0.0 ± 0.23	
	Day 9	0.0 ± 0.00	0.0 ± 0.00	0.0 ± 0.00	0.0 ± 0.00	
	Δ	0.0 ± 0.00	0.0 ± 0.00	0.0 ± 0.00	0.0 ± 0.00	
Runny nose of tearing	Baseline	0.0 ± 0.00	0.0 ± 0.00	0.0 ± 0.00	0.0 ± 0.00	
	Day 7	0.0 ± 0.00	0.0 ± 0.00	0.0 ± 0.00	0.0 ± 0.16	
	Δ	0.0 ± 0.00	0.0 ± 0.00	0.0 ± 0.00	0.0 ± 0.16	
	Day 9	0.0 ± 0.00	0.0 ± 0.00	0.0 ± 0.00	0.0 ± 0.00	
	Δ	0.0 ± 0.00	0.0 ± 0.00	0.0 ± 0.00	0.0 ± 0.00	
GI upset	Baseline	0.0 ± 0.00	0.0 ± 0.00	0.0 ± 0.00	0.0 ± 0.00	
	Day 7	0.0 ± 0.00	0.0 ± 0.00	0.0 ± 0.16	0.0 ± 0.16	
	Δ	0.0 ± 0.00	0.0 ± 0.00	0.0 ± 0.16	0.0 ± 0.16	
	Day 9	0.0 ± 0.00	0.0 ± 0.00	0.0 ± 0.16	0.0 ± 0.16	
	Δ	0.0 ± 0.00	0.0 ± 0.00	0.0 ± 0.16	0.0 ± 0.00	
Tremor	Baseline	0.0 ± 0.00	0.0 ± 0.00	0.0 ± 0.00	0.0 ± 0.00	
	Day 7	0.0 ± 0.00	0.0 ± 0.00	0.0 ± 0.00	0.0 ± 0.00	
	Δ	0.0 ± 0.00	0.0 ± 0.00	0.0 ± 0.00	0.0 ± 0.00	
	Day 9	0.0 ± 0.00	0.0 ± 0.00	0.0 ± 0.32	0.0 ± 0.32	
	Δ	0.0 ± 0.00	0.0 ± 0.00	0.1 ± 0.32	0.1 ± 0.32	
Yawning	Baseline	0.0 ± 0.00	0.0 ± 0.00	0.0 ± 0.00	0.0 ± 0.00	
	Day 7	0.0 ± 0.00	0.0 ± 0.00	0.0 ± 0.16	0.0 ± 0.39	
	Δ	0.0 ± 0.00	0.0 ± 0.00	0.0 ± 0.16	0.0 ± 0.39	
	Day 9	0.0 ± 0.00	0.0 ± 0.00	0.0 ± 0.00	0.0 ± 0.00	
	Δ	0.0 ± 0.00	0.0 ± 0.00	0.0 ± 0.00	0.0 ± 0.00	
Anxiety or irritability	Baseline	0.0 ± 0.00	0.0 ± 0.00	0.0 ± 0.00	0.0 ± 0.00	
	Day 7	0.0 ± 0.16	0.0 ± 0.16	0.0 ± 0.00	0.0 ± 0.16	
	Δ	0.0 ± 0.16	0.0 ± 0.16	0.0 ± 0.00	0.0 ± 0.16	
	Day 9	0.0 ± 0.00	0.0 ± 0.00	0.0 ± 0.16	0.0 ± 0.00	
	Δ	0.0 ± 0.00	0.0 ± 0.00	0.0 ± 0.16	0.0 ± 0.00	
Gooseflesh skin	Baseline	0.0 ± 0.00	0.0 ± 0.00	0.0 ± 0.00	0.0 ± 0.00	
	Day 7	0.0 ± 0.00	0.0 ± 0.00	0.0 ± 0.00	0.0 ± 0.00	
	Δ	0.0 ± 0.00	0.0 ± 0.00	0.0 ± 0.00	0.0 ± 0.00	
	Day 9	0.0 ± 0.00	0.0 ± 0.00	0.0 ± 0.00	0.0 ± 0.00	
	Δ	0.0 ± 0.00	0.0 ± 0.00	0.0 ± 0.00	0.0 ± 0.00	
Total score	Baseline	0.0 ± 0.16	0.0 ± 0.00	0.2 ± 0.36	0.1 ± 0.23	
	Day 7	0.1 ± 0.27	0.1 ± 0.41	0.3 ± 0.76	0.6 ± 0.98	
	Δ	0.1 ± 0.32	0.1 ± 0.41	0.2 ± 0.74	0.5 ± 1.01†*	
	Day 9	0.1 ± 0.22	0.0 ± 0.16	0.2 ± 0.61	0.2 ± 0.49	
	Δ	0.0 ± 0.16	0.0 ± 0.16	0.1 ± 0.68	0.2 ± 0.51	

**Table 14:** Change From Baseline to Day 6 in SOWS Values are mean ± SD. At baseline, n = 39, 40, 40, and 38 for placebo, low-dosage (LD), mid-dosage (MD), and high-dosage (HD) groups, respectively. At day 7 and day 8, n = 39, 39, 40, and 38 for placebo, low-dosage, mid-dosage, and high-dosage groups, respectively FFCT, Feel Free® Classic Tonic; Δ, change from baseline; SOWS, Subjective Opiate Withdrawal Scale

			FFCT Dosage Levels		
SOWS Score	Study Visit	Placebo Period	LD Period (15 mL)	MD Period (15 mL + 15 mL)	HD Period (30 mL + 30 mL)
Anxious	Baseline	0.1 ± 0.22	0.0 ± 0.00	0.0 ± 0.00	0.0 ± 0.00
	Day 7	0.1 ± 0.22	0.1 ± 0.48	0.0 ± 0.16	0.1 ± 0.32
	Δ	0.0 ± 0.23	0.1 ± 0.48	0.0 ± 0.16	0.1 ± 0.32
	Day 9	0.0 ± 0.00	0.1 ± 0.32	0.0 ± 0.16	0.0 ± 0.00
	Δ	-0.1 ± 0.22	0.1 ± 0.32	0.0 ± 0.16	0.0 ± 0.00
Bone and muscle aches	Baseline	0.0 ± 0.16	0.0 ± 0.00	0.0 ± 0.00	0.0 ± 0.00
	Day 7	0.0 ± 0.00	0.0 ± 0.00	0.0 ± 0.00	0.1 ± 0.32
	Δ	0.0 ± 0.16	0.0 ± 0.00	0.0 ± 0.00	0.1 ± 0.32
	Day 9	0.0 ± 0.00	0.0 ± 0.00	0.0 ± 0.00	0.0 ± 0.00
	Δ	0.0 ± 0.16	0.0 ± 0.00	0.0 ± 0.00	0.0 ± 0.00
Restless	Baseline	0.0 ± 0.16	0.0 ± 0.00	0.0 ± 0.00	0.0 ± 0.00
	Day 7	0.0 ± 0.00	0.1 ± 0.32	0.0 ± 0.00	0.0 ± 0.00
	Δ	0.0 ± 0.16	0.1 ± 0.32	0.0 ± 0.00	0.0 ± 0.00
	Day 9	0.0 ± 0.00	0.0 ± 0.00	0.0 ± 0.00	0.0 ± 0.00
	Δ	0.0 ± 0.16	0.0 ± 0.00	0.0 ± 0.00	0.0 ± 0.00
Nauseous	Baseline	0.0 ± 0.16	0.0 ± 0.00	0.0 ± 0.00	0.0 ± 0.00
	Day 7	0.0 ± 0.00	0.0 ± 0.00	0.0 ± 0.16	0.0 ± 0.16
	Δ	0.0 ± 0.16	0.0 ± 0.00	0.0 ± 0.16	0.0 ± 0.16
	Day 9	0.0 ± 0.00	0.0 ± 0.00	0.0 ± 0.16	0.0 ± 0.00
	Δ	0.0 ± 0.16	0.0 ± 0.00	0.0 ± 0.16	0.0 ± 0.00
Vomiting	Baseline	0.0 ± 0.00	0.0 ± 0.00	0.0 ± 0.00	0.0 ± 0.00
	Day 7	0.0 ± 0.00	0.0 ± 0.00	0.0 ± 0.00	0.0 ± 0.00
	Δ	0.0 ± 0.00	0.0 ± 0.00	0.0 ± 0.00	0.0 ± 0.00
	Day 9	0.0 ± 0.00	0.0 ± 0.00	0.0 ± 0.00	0.0 ± 0.00
	Δ	0.0 ± 0.00	0.0 ± 0.00	0.0 ± 0.00	0.0 ± 0.00
Muscle twitch	Baseline	0.0 ± 0.16	0.0 ± 0.00	0.0 ± 0.00	0.0 ± 0.00
	Day 7	0.0 ± 0.00	0.0 ± 0.00	0.0 ± 0.00	0.0 ± 0.00
	Δ	0.0 ± 0.16	0.0 ± 0.00	0.0 ± 0.00	0.0 ± 0.00
	Day 9	0.0 ± 0.00	0.0 ± 0.00	0.0 ± 0.00	0.0 ± 0.00
	Δ	0.0 ± 0.16	0.0 ± 0.00	0.0 ± 0.00	0.0 ± 0.00
Cramps in the stomach	Baseline	0.0 ± 0.16	0.0 ± 0.00	0.0 ± 0.00	0.0 ± 0.00
	Day 7	0.0 ± 0.00	0.0 ± 0.00	0.0 ± 0.16	0.0 ± 0.00
	Δ	0.0 ± 0.16	0.0 ± 0.00	0.0 ± 0.16	0.0 ± 0.00
	Day 9	0.0 ± 0.00	0.0 ± 0.00	0.0 ± 0.16	0.0 ± 0.00
	Δ	0.0 ± 0.16	0.0 ± 0.00	0.0 ± 0.16	0.0 ± 0.00
Feel like shooting up	Baseline	0.0 ± 0.00	0.0 ± 0.00	0.0 ± 0.00	0.0 ± 0.00
	Day 7	0.0 ± 0.00	0.0 ± 0.00	0.0 ± 0.00	0.0 ± 0.00
	Δ	0.0 ± 0.00	0.0 ± 0.00	0.0 ± 0.00	0.0 ± 0.00
	Day 9	0.0 ± 0.00	0.0 ± 0.00	0.0 ± 0.00	0.0 ± 0.00
	Δ	0.0 ± 0.00	0.0 ± 0.00	0.0 ± 0.00	0.0 ± 0.00
Yawning	Baseline	0.0 ± 0.16	0.0 ± 0.00	0.0 ± 0.00	0.0 ± 0.00
	Day 7	0.0 ± 0.16	0.1 ± 0.35	0.1 ± 0.50	0.1 ± 0.36
	Δ	0.0 ± 0.23	0.1 ± 0.35	0.1 ± 0.50	0.1 ± 0.36
	Day 9	0.0 ± 0.00	0.0 ± 0.00	0.0 ± 0.16	0.0 ± 0.00
	Δ	0.0 ± 0.16	0.0 ± 0.00	0.0 ± 0.16	0.0 ± 0.00
Perspiring	Baseline	0.0 ± 0.16	0.0 ± 0.00	0.0 ± 0.00	0.0 ± 0.00
	Day 7	0.0 ± 0.00	0.0 ± 0.16	0.1 ± 0.22	0.0 ± 0.00
	Δ	0.0 ± 0.16	0.0 ± 0.16	0.1 ± 0.22	0.0 ± 0.00
	Day 9	0.0 ± 0.00	0.1 ± 0.32	0.0 ± 0.16	0.0 ± 0.00
	Δ	0.0 ± 0.16	0.1 ± 0.32	0.0 ± 0.16	0.0 ± 0.00
Eyes tearing	Baseline	0.0 ± 0.00	0.0 ± 0.00	0.0 ± 0.00	0.0 ± 0.00
	Day 7	0.0 ± 0.00	0.0 ± 0.16	0.0 ± 0.00	0.0 ± 0.00
	Δ	0.0 ± 0.00	0.0 ± 0.16	0.0 ± 0.00	0.0 ± 0.00
	Day 9	0.0 ± 0.00	0.0 ± 0.00	0.0 ± 0.00	0.0 ± 0.00
	Δ	0.0 ± 0.00	0.0 ± 0.00	0.0 ± 0.00	0.0 ± 0.00
Runny nose	Baseline	0.0 ± 0.00	0.0 ± 0.00	0.0 ± 0.00	0.0 ± 0.00
	Day 7	0.0 ± 0.00	0.0 ± 0.00	0.0 ± 0.00	0.0 ± 0.16
	Δ	0.0 ± 0.00	0.0 ± 0.00	0.0 ± 0.00	0.0 ± 0.16
	Day 9	0.0 ± 0.00	0.0 ± 0.16	0.0 ± 0.00	0.0 ± 0.00
	Δ	0.0 ± 0.00	0.0 ± 0.16	0.0 ± 0.00	0.0 ± 0.00
Gooseflesh	Baseline	0.0 ± 0.00	0.0 ± 0.00	0.0 ± 0.00	0.0 ± 0.00
	Day 7	0.0 ± 0.00	0.0 ± 0.00	0.0 ± 0.00	0.0 ± 0.00
	Δ	0.0 ± 0.00	0.0 ± 0.00	0.0 ± 0.00	0.0 ± 0.00
	Day 9	0.0 ± 0.00	0.0 ± 0.00	0.0 ± 0.00	0.0 ± 0.00
	Δ	0.0 ± 0.00	0.0 ± 0.00	0.0 ± 0.00	0.0 ± 0.00
Shaking	Baseline	0.0 ± 0.16	0.0 ± 0.00	0.0 ± 0.00	0.0 ± 0.00
	Day 7	0.0 ± 0.00	0.0 ± 0.16	0.0 ± 0.16	0.0 ± 0.16
	Δ	0.0 ± 0.16	0.0 ± 0.16	0.0 ± 0.16	0.0 ± 0.16
	Day 9	0.0 ± 0.00	0.0 ± 0.00	0.0 ± 0.00	0.0 ± 0.00
	Δ	0.0 ± 0.16	0.0 ± 0.00	0.0 ± 0.00	0.0 ± 0.00
Hot flashes	Baseline	0.0 ± 0.00	0.0 ± 0.00	0.0 ± 0.00	0.0 ± 0.00
	Day 7	0.0 ± 0.00	0.0 ± 0.00	0.0 ± 0.00	0.0 ± 0.00
	Δ	0.0 ± 0.00	0.0 ± 0.00	0.0 ± 0.00	0.0 ± 0.00
	Day 9	0.0 ± 0.00	0.0 ± 0.00	0.0 ± 0.16	0.0 ± 0.00
	Δ	0.0 ± 0.00	0.0 ± 0.00	0.0 ± 0.16	0.0 ± 0.00
Cold flashes	Baseline	0.0 ± 0.16	0.0 ± 0.00	0.0 ± 0.00	0.0 ± 0.00
	Day 7	0.0 ± 0.16	0.0 ± 0.16	0.0 ± 0.00	0.0 ± 0.00
	Δ	0.0 ± 0.00	0.0 ± 0.16	0.0 ± 0.00	0.0 ± 0.00
	Day 9	0.0 ± 0.00	0.0 ± 0.00	0.0 ± 0.00	0.0 ± 0.00
	Δ	0.0 ± 0.16	0.0 ± 0.00	0.0 ± 0.00	0.0 ± 0.00
Total score	Baseline	0.3 ± 1.28	0.0 ± 0.00	0.0 ± 0.00	0.0 ± 0.00
	Day 7	0.1 ± 0.38	0.3 ± 1.36	0.3 ± 1.16	0.3 ± 1.03
	Δ	-0.2 ± 1.19	0.3 ± 1.36	0.3 ± 1.16	0.3 ± 1.03
	Day 9	0.0 ± 0.00	0.1 ± 0.80	0.2 ± 0.95	0.0 ± 0.00
	Δ	-0.3 ± 1.28	0.1 ± 0.80	0.2 ± 0.95	0.0 ± 0.00

Table 15 and Table 16 show the analysis of a sub-group for COWS and SOWS, respectively.

**Table 15:** Change From Baseline to Day 6 in COWS (Sub-group Analysis) Values are mean ± SD. At baseline, n = 39, 40, 40, and 38 for placebo, low-dosage (LD), mid-dosage (MD), and high-dosage (HD) groups, respectively. At day 7 and day 8, n = 39, 39, 40, and 38 for placebo, low-dosage, mid-dosage, and high-dosage groups, respectively FFCT, Feel Free® Classic Tonic; GI, gastrointestinal; Δ, change from baseline; COWS, Clinical Opiate Withdrawal Scale

			FFCT Dosage Levels		
COWS Score	Study Visit	Placebo Period	LD Period (15 mL)	MD Period (15 mL + 15 mL)	HD Period (30 mL + 30 mL)
Resting pulse rate	Baseline	0.0 ± 0.00	0.0 ± 0.00	0.1 ± 0.33	0.0 ± 0.20
	Day 7	0.1 ± 0.28	0.0 ± 0.00	0.1 ± 0.33	0.3 ± 0.48
	Δ	0.1 ± 0.28	0.0 ± 0.00	0.0 ± 0.41	0.3 ± 0.54
	Day 9	0.0 ± 0.20	0.0 ± 0.00	0.0 ± 0.20	0.2 ± 0.41
	Δ	0.0 ± 0.20	0.0 ± 0.00	-0.1 ± 0.28	0.2 ± 0.37
Sweating	Baseline	0.0 ± 0.00	0.0 ± 0.00	0.0 ± 0.00	0.0 ± 0.00
	Day 7	0.0 ± 0.00	0.0 ± 0.00	0.0 ± 0.00	0.0 ± 0.00
	Δ	0.0 ± 0.00	0.0 ± 0.00	0.0 ± 0.00	0.0 ± 0.00
	Day 9	0.0 ± 0.00	0.0 ± 0.00	0.0 ± 0.00	0.0 ± 0.00
	Δ	0.0 ± 0.00	0.0 ± 0.00	0.0 ± 0.00	0.0 ± 0.00
Restlessness	Baseline	0.0 ± 0.00	0.0 ± 0.00	0.0 ± 0.00	0.0 ± 0.00
	Day 7	0.0 ± 0.00	0.0 ± 0.00	0.0 ± 0.00	0.0 ± 0.20
	Δ	0.0 ± 0.00	0.0 ± 0.00	0.0 ± 0.00	0.0 ± 0.20
	Day 9	0.0 ± 0.00	0.0 ± 0.00	0.0 ± 0.00	0.0 ± 0.00
	Δ	0.0 ± 0.00	0.0 ± 0.00	0.0 ± 0.00	0.0 ± 0.00
Pupil size	Baseline	0.0 ± 0.00	0.0 ± 0.00	0.0 ± 0.00	0.0 ± 0.00
	Day 7	0.0 ± 0.00	0.0 ± 0.00	0.0 ± 0.00	0.0 ± 0.00
	Δ	0.0 ± 0.00	0.0 ± 0.00	0.0 ± 0.00	0.0 ± 0.00
	Day 9	0.0 ± 0.00	0.0 ± 0.00	0.0 ± 0.00	0.0 ± 0.00
	Δ	0.0 ± 0.00	0.0 ± 0.00	0.0 ± 0.00	0.0 ± 0.00
Bone or joint aches	Baseline	0.0 ± 0.00	0.0 ± 0.00	0.0 ± 0.00	0.0 ± 0.00
	Day 7	0.0 ± 0.00	0.0 ± 0.00	0.0 ± 0.00	0.1 ± 0.28
	Δ	0.0 ± 0.00	0.0 ± 0.00	0.0 ± 0.00	0.1 ± 0.28
	Day 9	0.0 ± 0.00	0.0 ± 0.00	0.0 ± 0.00	0.0 ± 0.00
	Δ	0.0 ± 0.00	0.0 ± 0.00	0.0 ± 0.00	0.0 ± 0.00
Runny nose of tearing	Baseline	0.0 ± 0.00	0.0 ± 0.00	0.0 ± 0.00	0.0 ± 0.00
	Day 7	0.0 ± 0.00	0.0 ± 0.00	0.0 ± 0.00	0.0 ± 0.20
	Δ	0.0 ± 0.00	0.0 ± 0.00	0.0 ± 0.00	0.0 ± 0.20
	Day 9	0.0 ± 0.00	0.0 ± 0.00	0.0 ± 0.00	0.0 ± 0.00
	Δ	0.0 ± 0.00	0.0 ± 0.00	0.0 ± 0.00	0.0 ± 0.00
GI upset	Baseline	0.0 ± 0.00	0.0 ± 0.00	0.0 ± 0.00	0.0 ± 0.00
	Day 7	0.0 ± 0.00	0.0 ± 0.00	0.0 ± 0.00	0.0 ± 0.20
	Δ	0.0 ± 0.00	0.0 ± 0.00	0.0 ± 0.00	0.0 ± 0.20
	Day 9	0.0 ± 0.00	0.0 ± 0.00	0.0 ± 0.00	0.0 ± 0.00
	Δ	0.0 ± 0.00	0.0 ± 0.00	0.0 ± 0.00	0.0 ± 0.00
Tremor	Baseline	0.0 ± 0.00	0.0 ± 0.00	0.0 ± 0.00	0.0 ± 0.00
	Day 7	0.0 ± 0.00	0.0 ± 0.00	0.0 ± 0.00	0.0 ± 0.00
	Δ	0.0 ± 0.00	0.0 ± 0.00	0.0 ± 0.00	0.0 ± 0.00
	Day 9	0.0 ± 0.00	0.0 ± 0.00	0.0 ± 0.00	0.1 ± 0.40
	Δ	0.0 ± 0.00	0.0 ± 0.00	0.0 ± 0.00	0.1 ± 0.40
Yawning	Baseline	0.0 ± 0.00	0.0 ± 0.00	0.0 ± 0.00	0.0 ± 0.00
	Day 7	0.0 ± 0.00	0.0 ± 0.00	0.0 ± 0.00	0.2 ± 0.47
	Δ	0.0 ± 0.00	0.0 ± 0.00	0.0 ± 0.00	0.2 ± 0.47
	Day 9	0.0 ± 0.00	0.0 ± 0.00	0.0 ± 0.00	0.0 ± 0.00
	Δ	0.0 ± 0.00	0.0 ± 0.00	0.0 ± 0.00	0.0 ± 0.00
Anxiety or irritability	Baseline	0.0 ± 0.00	0.0 ± 0.00	0.0 ± 0.00	0.0 ± 0.00
	Day 7	0.0 ± 0.00	0.0 ± 0.00	0.0 ± 0.00	0.0 ± 0.20
	Δ	0.0 ± 0.00	0.0 ± 0.00	0.0 ± 0.00	0.0 ± 0.20
	Day 9	0.0 ± 0.00	0.0 ± 0.00	0.0 ± 0.00	0.0 ± 0.00
	Δ	0.0 ± 0.00	0.0 ± 0.00	0.0 ± 0.00	0.0 ± 0.00
Gooseflesh skin	Baseline	0.0 ± 0.00	0.0 ± 0.00	0.0 ± 0.00	0.0 ± 0.00
	Day 7	0.0 ± 0.00	0.0 ± 0.00	0.0 ± 0.00	0.0 ± 0.00
	Δ	0.0 ± 0.00	0.0 ± 0.00	0.0 ± 0.00	0.0 ± 0.00
	Day 9	0.0 ± 0.00	0.0 ± 0.00	0.0 ± 0.00	0.0 ± 0.00
	Δ	0.0 ± 0.00	0.0 ± 0.00	0.0 ± 0.00	0.0 ± 0.00
Total score	Baseline	0.0 ± 0.00	0.0 ± 0.00	0.1 ± 0.33	0.0 ± 0.20
	Day 7	0.1 ± 0.33	0.1 ± 0.40	0.2 ± 0.83	0.8 ± 1.13
	Δ	0.1 ± 0.33	0.1 ± 0.40	0.1 ± 0.88	0.7 ± 1.17
	Day 9	0.0 ± 0.20	0.0 ± 0.20	0.2 ± 0.72	0.3 ± 0.54
	Δ	0.0 ± 0.20	0.0 ± 0.20	0.1 ± 0.78	0.2 ± 0.52

**Table 16:** Change From Baseline to Day 6 in SOWS (Sub-group Analysis) Values are mean ± SD. At baseline, n = 39, 40, 40, and 38 for placebo, low-dosage (LD), mid-dosage (MD), and high-dosage (HD) groups, respectively. At day 7 and day 8, n = 39, 39, 40, and 38 for placebo, low-dosage, mid-dosage, and high-dosage groups, respectively FFCT, Feel Free® Classic Tonic; Δ, change from baseline; SOWS, Subjective Opiate Withdrawal Scale

			FFCT Dosage Levels		
SOWS Score	Study Visit	Placebo Period	LD Period (15 mL)	MD Period (15 mL + 15 mL)	HD Period (30 mL + 30 mL)
Anxious	Baseline	0.0 ± 0.20	0.0 ± 0.00	0.0 ± 0.00	0.0 ± 0.00
	Day 7	0.1 ± 0.28	0.0 ± 0.00	0.0 ± 0.20	0.1 ± 0.40
	Δ	0.0 ± 0.20	0.0 ± 0.00	0.0 ± 0.20	0.1 ± 0.40
	Day 9	0.0 ± 0.00	0.1 ± 0.40	0.0 ± 0.20	0.0 ± 0.00
	Δ	0.0 ± 0.20	0.1 ± 0.40	0.0 ± 0.20	0.0 ± 0.00
Bone and muscle aches	Baseline	0.0 ± 0.00	0.0 ± 0.00	0.0 ± 0.00	0.0 ± 0.00
	Day 7	0.0 ± 0.00	0.0 ± 0.00	0.0 ± 0.00	0.1 ± 0.40
	Δ	0.0 ± 0.00	0.0 ± 0.00	0.0 ± 0.00	0.1 ± 0.40
	Day 9	0.0 ± 0.00	0.0 ± 0.00	0.0 ± 0.00	0.0 ± 0.00
	Δ	0.0 ± 0.00	0.0 ± 0.00	0.0 ± 0.00	0.0 ± 0.00
Restless	Baseline	0.0 ± 0.00	0.0 ± 0.00	0.0 ± 0.00	0.0 ± 0.00
	Day 7	0.0 ± 0.00	0.0 ± 0.00	0.0 ± 0.00	0.0 ± 0.00
	Δ	0.0 ± 0.00	0.0 ± 0.00	0.0 ± 0.00	0.0 ± 0.00
	Day 9	0.0 ± 0.00	0.0 ± 0.00	0.0 ± 0.00	0.0 ± 0.00
	Δ	0.0 ± 0.00	0.0 ± 0.00	0.0 ± 0.00	0.0 ± 0.00
Nauseous	Baseline	0.0 ± 0.00	0.0 ± 0.00	0.0 ± 0.00	0.0 ± 0.00
	Day 7	0.0 ± 0.00	0.0 ± 0.00	0.0 ± 0.20	0.0 ± 0.20
	Δ	0.0 ± 0.00	0.0 ± 0.00	0.0 ± 0.20	0.0 ± 0.20
	Day 9	0.0 ± 0.00	0.0 ± 0.00	0.0 ± 0.20	0.0 ± 0.00
	Δ	0.0 ± 0.00	0.0 ± 0.00	0.0 ± 0.20	0.0 ± 0.00
Vomiting	Baseline	0.0 ± 0.00	0.0 ± 0.00	0.0 ± 0.00	0.0 ± 0.00
	Day 7	0.0 ± 0.00	0.0 ± 0.00	0.0 ± 0.00	0.0 ± 0.00
	Δ	0.0 ± 0.00	0.0 ± 0.00	0.0 ± 0.00	0.0 ± 0.00
	Day 9	0.0 ± 0.00	0.0 ± 0.00	0.0 ± 0.00	0.0 ± 0.00
	Δ	0.0 ± 0.00	0.0 ± 0.00	0.0 ± 0.00	0.0 ± 0.00
Muscle twitch	Baseline	0.0 ± 0.00	0.0 ± 0.00	0.0 ± 0.00	0.0 ± 0.00
	Day 7	0.0 ± 0.00	0.0 ± 0.00	0.0 ± 0.00	0.0 ± 0.00
	Δ	0.0 ± 0.00	0.0 ± 0.00	0.0 ± 0.00	0.0 ± 0.00
	Day 9	0.0 ± 0.00	0.0 ± 0.00	0.0 ± 0.00	0.0 ± 0.00
	Δ	0.0 ± 0.00	0.0 ± 0.00	0.0 ± 0.00	0.0 ± 0.00
Cramps in the stomach	Baseline	0.0 ± 0.00	0.0 ± 0.00	0.0 ± 0.00	0.0 ± 0.00
	Day 7	0.0 ± 0.00	0.0 ± 0.00	0.0 ± 0.20	0.0 ± 0.00
	Δ	0.0 ± 0.00	0.0 ± 0.00	0.0 ± 0.20	0.0 ± 0.00
	Day 9	0.0 ± 0.00	0.0 ± 0.00	0.0 ± 0.20	0.0 ± 0.00
	Δ	0.0 ± 0.00	0.0 ± 0.00	0.0 ± 0.20	0.0 ± 0.00
Feel like shooting up	Baseline	0.0 ± 0.00	0.0 ± 0.00	0.0 ± 0.00	0.0 ± 0.00
	Day 7	0.0 ± 0.00	0.0 ± 0.00	0.0 ± 0.00	0.0 ± 0.00
	Δ	0.0 ± 0.00	0.0 ± 0.00	0.0 ± 0.00	0.0 ± 0.00
	Day 9	0.0 ± 0.00	0.0 ± 0.00	0.0 ± 0.00	0.0 ± 0.00
	Δ	0.0 ± 0.00	0.0 ± 0.00	0.0 ± 0.00	0.0 ± 0.00
Yawning	Baseline	0.0 ± 0.00	0.0 ± 0.00	0.0 ± 0.00	0.0 ± 0.00
	Day 7	0.0 ± 0.00	0.0 ± 0.00	0.1 ± 0.60	0.1 ± 0.44
	Δ	0.0 ± 0.00	0.0 ± 0.00	0.1 ± 0.60	0.1 ± 0.44
	Day 9	0.0 ± 0.00	0.0 ± 0.00	0.0 ± 0.20	0.0 ± 0.00
	Δ	0.0 ± 0.00	0.0 ± 0.00	0.0 ± 0.20	0.0 ± 0.00
Perspiring	Baseline	0.0 ± 0.20	0.0 ± 0.00	0.0 ± 0.00	0.0 ± 0.00
	Day 7	0.0 ± 0.00	0.0 ± 0.20	0.1 ± 0.28	0.0 ± 0.00
	Δ	0.0 ± 0.20	0.0 ± 0.20	0.1 ± 0.28	0.0 ± 0.00
	Day 9	0.0 ± 0.00	0.1 ± 0.40	0.0 ± 0.20	0.0 ± 0.00
	Δ	0.0 ± 0.20	0.1 ± 0.40	0.0 ± 0.20	0.0 ± 0.00
Eyes tearing	Baseline	0.0 ± 0.00	0.0 ± 0.00	0.0 ± 0.00	0.0 ± 0.00
	Day 7	0.0 ± 0.00	0.0 ± 0.20	0.0 ± 0.00	0.0 ± 0.00
	Δ	0.0 ± 0.00	0.0 ± 0.20	0.0 ± 0.00	0.0 ± 0.00
	Day 9	0.0 ± 0.00	0.0 ± 0.00	0.0 ± 0.00	0.0 ± 0.00
	Δ	0.0 ± 0.00	0.0 ± 0.00	0.0 ± 0.00	0.0 ± 0.00
Runny nose	Baseline	0.0 ± 0.00	0.0 ± 0.00	0.0 ± 0.00	0.0 ± 0.00
	Day 7	0.0 ± 0.00	0.0 ± 0.00	0.0 ± 0.00	0.0 ± 0.20
	Δ	0.0 ± 0.00	0.0 ± 0.00	0.0 ± 0.00	0.0 ± 0.20
	Day 9	0.0 ± 0.00	0.0 ± 0.20	0.0 ± 0.00	0.0 ± 0.00
	Δ	0.0 ± 0.00	0.0 ± 0.20	0.0 ± 0.00	0.0 ± 0.00
Gooseflesh	Baseline	0.0 ± 0.00	0.0 ± 0.00	0.0 ± 0.00	0.0 ± 0.00
	Day 7	0.0 ± 0.00	0.0 ± 0.00	0.0 ± 0.00	0.0 ± 0.00
	Δ	0.0 ± 0.00	0.0 ± 0.00	0.0 ± 0.00	0.0 ± 0.00
	Day 9	0.0 ± 0.00	0.0 ± 0.00	0.0 ± 0.00	0.0 ± 0.00
	Δ	0.0 ± 0.00	0.0 ± 0.00	0.0 ± 0.00	0.0 ± 0.00
Shaking	Baseline	0.0 ± 0.00	0.0 ± 0.00	0.0 ± 0.00	0.0 ± 0.00
	Day 7	0.0 ± 0.00	0.0 ± 0.00	0.0 ± 0.20	0.0 ± 0.20
	Δ	0.0 ± 0.20	0.0 ± 0.00	0.0 ± 0.20	0.0 ± 0.20
	Day 9	0.0 ± 0.00	0.0 ± 0.00	0.0 ± 0.00	0.0 ± 0.00
	Δ	0.0 ± 0.20	0.0 ± 0.00	0.0 ± 0.00	0.0 ± 0.00
Hot flashes	Baseline	0.0 ± 0.00	0.0 ± 0.00	0.0 ± 0.00	0.0 ± 0.00
	Day 7	0.0 ± 0.00	0.0 ± 0.00	0.0 ± 0.00	0.0 ± 0.00
	Δ	0.0 ± 0.20	0.0 ± 0.20	0.0 ± 0.20	0.0 ± 0.20
	Day 9	0.0 ± 0.00	0.0 ± 0.00	0.0 ± 0.00	0.0 ± 0.00
	Δ	0.0 ± 0.20	0.0 ± 0.20	0.0 ± 0.20	0.0 ± 0.20
Cold flashes	Baseline	0.0 ± 0.20	0.0 ± 0.00	0.0 ± 0.00	0.0 ± 0.00
	Day 7	0.0 ± 0.20	0.0 ± 0.20	0.0 ± 0.00	0.0 ± 0.00
	Δ	0.0 ± 0.00	0.0 ± 0.20	0.0 ± 0.00	0.0 ± 0.00
	Day 9	0.0 ± 0.00	0.0 ± 0.00	0.0 ± 0.00	0.0 ± 0.00
	Δ	0.0 ± 0.20	0.0 ± 0.00	0.0 ± 0.00	0.0 ± 0.00
Total score	Baseline	0.2 ± 0.80	0.0 ± 0.00	0.0 ± 0.00	0.0 ± 0.00
	Day 7	0.1 ± 0.44	0.1 ± 0.60	0.4 ± 1.44	0.4 ± 1.26
	Δ	0.0 ± 0.45	0.1 ± 0.60	0.4 ± 1.44	0.4 ± 1.26
	Day 9	0.0 ± 0.00	0.2 ± 1.00	0.2 ± 1.20	0.0 ± 0.00
	Δ	-0.2 ± 0.80	0.2 ± 1.00	0.2 ± 1.20	0.0 ± 0.00

Table 17 shows the summary of adverse events by system organ class and preferred term.

**Table 17:** Adverse Events by System Organ Class and Preferred Term HD, high dosage; LD, low dosage; MD, mid-dosage; N, number of participants

		Placebo Period		LD Period		MD Period		HD Period		Total		
System Organ Class	Preferred Term	Participants (N = 39)	Events	Participants (N = 40)	Events	Participants (N = 40)	Events	Participants (N = 38)	Events	Participants (N = 54)	Events	
												Overall		9 (23.1%)	14 (100%)	6 (15.0%)	17 (100%)	10 (25.0%)	18 (100%)	17 (44.7%)	90 (100%)	30 (55.6%)	139 (100%)	
Cardiac disorders	Tachycardia	0 (0.0%)	0 (0.0%)	0 (0.0%)	0 (0.0%)	1 (2.5%)	1 (5.6%)	0 (0.0%)	0 (0.0%)	1 (1.9%)	1 (0.7%)	
Gastrointestinal disorders	Overall	0 (0.0%)	0 (0.0%)	1 (2.5%)	1 (5.9%)	3 (7.5%)	3 (16.7%)	13 (34.2%)	25 (27.8%)	15 (27.8%)	29 (20.9%)	
	Aphthous ulcer	0 (0.0%)	0 (0.0%)	0 (0.0%)	0 (0.0%)	0 (0.0%)	0 (0.0%)	1 (2.6%)	1 (1.1%)	1 (1.9%)	1 (0.7%)	
	Constipation	0 (0.0%)	0 (0.0%)	0 (0.0%)	0 (0.0%)	1 (2.5%)	1 (5.6%)	2 (5.3%)	2 (2.2%)	3 (5.6%)	3 (2.2%)	
	Diarrhea	0 (0.0%)	0 (0.0%)	0 (0.0%)	0 (0.0%)	1 (2.5%)	1 (5.6%)	0 (0.0%)	0 (0.0%)	1 (1.9%)	1 (0.7%)	
	Dyspepsia	0 (0.0%)	0 (0.0%)	0 (0.0%)	0 (0.0%)	0 (0.0%)	0 (0.0%)	1 (2.6%)	3 (3.3%)	1 (1.9%)	3 (2.2%)	
	Infrequent bowel movements	0 (0.0%)	0 (0.0%)	0 (0.0%)	0 (0.0%)	0 (0.0%)	0 (0.0%)	1 (2.6%)	1 (1.1%)	1 (1.9%)	1 (0.7%)	
	Nausea	0 (0.0%)	0 (0.0%)	1 (2.5%)	1 (5.9%)	1 (2.5%)	1 (5.6%)	11 (28.9%)	13 (14.4%)	12 (22.2%)	15 (10.8%)	
	Vomiting	0 (0.0%)	0 (0.0%)	0 (0.0%)	0 (0.0%)	0 (0.0%)	0 (0.0%)	3 (7.9%)	5 (5.6%)	3 (5.6%)	5 (3.6%)	
General disorders and administration site conditions	Overall	1 (2.6%)	1 (7.1%)	2 (5.0%)	7 (41.2%)	0 (0.0%)	0 (0.0%)	8 (21.1%)	18 (20.0%)	10 (18.5%)	26 (18.7%)	
	Asthenia	0 (0.0%)	0 (0.0%)	1 (2.5%)	1 (5.9%)	0 (0.0%)	0 (0.0%)	0 (0.0%)	0 (0.0%)	1 (1.9%)	1 (0.7%)	
	Chest discomfort	1 (2.6%)	1 (7.1%)	0 (0.0%)	0 (0.0%)	0 (0.0%)	0 (0.0%)	0 (0.0%)	0 (0.0%)	1 (1.9%)	1 (0.7%)	
	Chills	0 (0.0%)	0 (0.0%)	1 (2.5%)	2 (11.8%)	0 (0.0%)	0 (0.0%)	0 (0.0%)	0 (0.0%)	1 (1.9%)	2 (1.4%)	
	Fatigue	0 (0.0%)	0 (0.0%)	1 (2.5%)	3 (17.6%)	0 (0.0%)	0 (0.0%)	8 (21.1%)	14 (15.6%)	9 (16.7%)	17 (12.2%)	
	Feeling hot	0 (0.0%)	0 (0.0%)	0 (0.0%)	0 (0.0%)	0 (0.0%)	0 (0.0%)	2 (5.3%)	3 (3.3%)	2 (3.7%)	3 (2.2%)	
	Feeling jittery	0 (0.0%)	0 (0.0%)	0 (0.0%)	0 (0.0%)	0 (0.0%)	0 (0.0%)	1 (2.6%)	1 (1.1%)	1 (1.9%)	1 (0.7%)	
	Pyrexia	0 (0.0%)	0 (0.0%)	1 (2.5%)	1 (5.9%)	0 (0.0%)	0 (0.0%)	0 (0.0%)	0 (0.0%)	1 (1.9%)	1 (0.7%)	
Infections and infestations	Overall	2 (5.1%)	2 (14.3%)	3 (7.5%)	3 (17.6%)	1 (2.5%)	1 (5.6%)	0 (0.0%)	0 (0.0%)	6 (11.1%)	6 (4.3%)	
	Conjunctivitis	0 (0.0%)	0 (0.0%)	0 (0.0%)	0 (0.0%)	1 (2.5%)	1 (5.6%)	0 (0.0%)	0 (0.0%)	1 (1.9%)	1 (0.7%)	
	Upper respiratory tract infection	1 (2.6%)	1 (7.1%)	3 (7.5%)	3 (17.6%)	0 (0.0%)	0 (0.0%)	0 (0.0%)	0 (0.0%)	4 (7.4%)	4 (2.9%)	
	Viral infection	1 (2.6%)	1 (7.1%)	0 (0.0%)	0 (0.0%)	0 (0.0%)	0 (0.0%)	0 (0.0%)	0 (0.0%)	1 (1.9%)	1 (0.7%)	
	Injury, poisoning, and procedural complications	1 (2.6%)	1 (7.1%)	0 (0.0%)	0 (0.0%)	0 (0.0%)	0 (0.0%)	1 (2.6%)	1 (1.1%)	1 (1.9%)	2 (1.4%)	
	Muscle strain	0 (0.0%)	0 (0.0%)	0 (0.0%)	0 (0.0%)	0 (0.0%)	0 (0.0%)	1 (2.6%)	1 (1.1%)	1 (1.9%)	1 (0.7%)	
	Thermal burn	1 (2.6%)	1 (7.1%)	0 (0.0%)	0 (0.0%)	0 (0.0%)	0 (0.0%)	0 (0.0%)	0 (0.0%)	1 (1.9%)	1 (0.7%)	
Investigations	Overall	1 (2.6%)	2 (14.3%)	0 (0.0%)	0 (0.0%)	2 (5.0%)	2 (11.1%)	0 (0.0%)	0 (0.0%)	3 (5.6%)	4 (2.9%)	
	Alanine aminotransferase increased	1 (2.6%)	1 (7.1%)	0 (0.0%)	0 (0.0%)	0 (0.0%)	0 (0.0%)	0 (0.0%)	0 (0.0%)	1 (1.9%)	1 (0.7%)	
	Aspartate aminotransferase increased	1 (2.6%)	1 (7.1%)	0 (0.0%)	0 (0.0%)	0 (0.0%)	0 (0.0%)	0 (0.0%)	0 (0.0%)	1 (1.9%)	1 (0.7%)	
	Blood pressure increased	0 (0.0%)	0 (0.0%)	0 (0.0%)	0 (0.0%)	1 (2.5%)	1 (5.6%)	0 (0.0%)	0 (0.0%)	1 (1.9%)	1 (0.7%)	
	Gamma-glutamyl transferase increased	0 (0.0%)	0 (0.0%)	0 (0.0%)	0 (0.0%)	1 (2.5%)	1 (5.6%)	0 (0.0%)	0 (0.0%)	1 (1.9%)	1 (0.7%)	
Metabolism and nutrition disorders	Overall	1 (2.6%)	1 (7.1%)	0 (0.0%)	0 (0.0%)	3 (7.5%)	4 (22.2%)	4 (10.5%)	4 (4.4%)	8 (14.8%)	9 (6.5%)	
	Decreased appetite	0 (0.0%)	0 (0.0%)	0 (0.0%)	0 (0.0%)	2 (5.0%)	2 (11.1%)	4 (10.5%)	4 (4.4%)	6 (11.1%)	6 (4.3%)	
	Hypochloremia	0 (0.0%)	0 (0.0%)	0 (0.0%)	0 (0.0%)	1 (2.5%)	1 (5.6%)	0 (0.0%)	0 (0.0%)	1 (1.9%)	1 (0.7%)	
	Hyponatremia	0 (0.0%)	0 (0.0%)	0 (0.0%)	0 (0.0%)	1 (2.5%)	1 (5.6%)	0 (0.0%)	0 (0.0%)	1 (1.9%)	1 (0.7%)	
	Impaired fasting glucose	1 (2.6%)	1 (7.1%)	0 (0.0%)	0 (0.0%)	0 (0.0%)	0 (0.0%)	0 (0.0%)	0 (0.0%)	1 (1.9%)	1 (0.7%)	
Musculoskeletal and connective tissue disorders	Overall	0 (0.0%)	0 (0.0%)	0 (0.0%)	0 (0.0%)	1 (2.5%)	1 (5.6%)	3 (7.9%)	4 (4.4%)	4 (7.4%)	5 (3.6%)	
	Back pain	0 (0.0%)	0 (0.0%)	0 (0.0%)	0 (0.0%)	0 (0.0%)	0 (0.0%)	2 (5.3%)	2 (2.2%)	2 (3.7%)	2 (1.4%)	
	Myalgia	0 (0.0%)	0 (0.0%)	0 (0.0%)	0 (0.0%)	0 (0.0%)	0 (0.0%)	1 (2.6%)	1 (1.1%)	1 (1.9%)	1 (0.7%)	
	Neck pain	0 (0.0%)	0 (0.0%)	0 (0.0%)	0 (0.0%)	1 (2.5%)	1 (5.6%)	0 (0.0%)	0 (0.0%)	1 (1.9%)	1 (0.7%)	
	Pain in extremity	0 (0.0%)	0 (0.0%)	0 (0.0%)	0 (0.0%)	0 (0.0%)	0 (0.0%)	1 (2.6%)	1 (1.1%)	1 (1.9%)	1 (0.7%)	
Nervous system disorders	Overall	4 (10.3%)	4 (28.6%)	3 (7.5%)	5 (29.4%)	3 (7.5%)	4 (22.2%)	11 (28.9%)	29 (32.2%)	17 (31.5%)	42 (30.2%)	
	Brain fog	0 (0.0%)	0 (0.0%)	0 (0.0%)	0 (0.0%)	0 (0.0%)	0 (0.0%)	1 (2.6%)	1 (1.1%)	1 (1.9%)	1 (0.7%)	
	Cognitive disorder	0 (0.0%)	0 (0.0%)	0 (0.0%)	0 (0.0%)	0 (0.0%)	0 (0.0%)	1 (2.6%)	1 (1.1%)	1 (1.9%)	1 (0.7%)	
	Disturbance in attention	0 (0.0%)	0 (0.0%)	0 (0.0%)	0 (0.0%)	0 (0.0%)	0 (0.0%)	2 (5.3%)	3 (3.3%)	2 (3.7%)	3 (2.2%)	
	Dizziness	0 (0.0%)	0 (0.0%)	0 (0.0%)	0 (0.0%)	0 (0.0%)	0 (0.0%)	4 (10.5%)	4 (4.4%)	4 (7.4%)	4 (2.9%)	
	Headache	4 (10.3%)	4 (28.6%)	3 (7.5%)	5 (29.4%)	3 (7.5%)	3 (16.7%)	11 (28.9%)	15 (16.7%)	17 (31.5%)	27 (19.4%)	
	Memory impairment	0 (0.0%)	0 (0.0%)	0 (0.0%)	0 (0.0%)	0 (0.0%)	0 (0.0%)	1 (2.6%)	1 (1.1%)	1 (1.9%)	1 (0.7%)	
	Paresthesia	0 (0.0%)	0 (0.0%)	0 (0.0%)	0 (0.0%)	1 (2.5%)	1 (5.6%)	0 (0.0%)	0 (0.0%)	1 (1.9%)	1 (0.7%)	
	Sensory disturbance	0 (0.0%)	0 (0.0%)	0 (0.0%)	0 (0.0%)	0 (0.0%)	0 (0.0%)	1 (2.6%)	1 (1.1%)	1 (1.9%)	1 (0.7%)	
	Somnolence	0 (0.0%)	0 (0.0%)	0 (0.0%)	0 (0.0%)	0 (0.0%)	0 (0.0%)	3 (7.9%)	3 (3.3%)	3 (5.6%)	3 (2.2%)	
Psychiatric disorders	Overall	0 (0.0%)	0 (0.0%)	1 (2.5%)	1 (5.9%)	2 (5.0%)	2 (11.1%)	1 (2.6%)	3 (3.3%)	4 (7.4%)	6 (4.3%)	
	Abnormal dreams	0 (0.0%)	0 (0.0%)	0 (0.0%)	0 (0.0%)	1 (2.5%)	1 (5.6%)	1 (2.6%)	1 (1.1%)	2 (3.7%)	2 (1.4%)	
	Depressed mood	0 (0.0%)	0 (0.0%)	1 (2.5%)	1 (5.9%)	0 (0.0%)	0 (0.0%)	0 (0.0%)	0 (0.0%)	1 (1.9%)	1 (0.7%)	
	Sleep disorder	0 (0.0%)	0 (0.0%)	0 (0.0%)	0 (0.0%)	1 (2.5%)	1 (5.6%)	1 (2.6%)	2 (2.2%)	2 (3.7%)	3 (2.2%)	
Respiratory, thoracic, and mediastinal disorders	Overall	3 (7.7%)	3 (21.4%)	0 (0.0%)	0 (0.0%)	0 (0.0%)	0 (0.0%)	0 (0.0%)	0 (0.0%)	3 (5.6%)	3 (2.2%)	
	Oropharyngeal pain	1 (2.6%)	1 (7.1%)	0 (0.0%)	0 (0.0%)	0 (0.0%)	0 (0.0%)	0 (0.0%)	0 (0.0%)	1 (1.9%)	1 (0.7%)	
	Rhinorrhea	1 (2.6%)	1 (7.1%)	0 (0.0%)	0 (0.0%)	0 (0.0%)	0 (0.0%)	0 (0.0%)	0 (0.0%)	1 (1.9%)	1 (0.7%)	
	Yawning	1 (2.6%)	1 (7.1%)	0 (0.0%)	0 (0.0%)	0 (0.0%)	0 (0.0%)	0 (0.0%)	0 (0.0%)	1 (1.9%)	1 (0.7%)	
Skin and subcutaneous tissue disorders	Overall	0 (0.0%)	0 (0.0%)	0 (0.0%)	0 (0.0%)	0 (0.0%)	0 (0.0%)	6 (15.8%)	6 (6.7%)	6 (11.1%)	6 (4.3%)	
	Hyperhidrosis	0 (0.0%)	0 (0.0%)	0 (0.0%)	0 (0.0%)	0 (0.0%)	0 (0.0%)	1 (2.6%)	1 (1.1%)	1 (1.9%)	1 (0.7%)	
	Pruritus	0 (0.0%)	0 (0.0%)	0 (0.0%)	0 (0.0%)	0 (0.0%)	0 (0.0%)	5 (13.2%)	5 (5.6%)	5 (9.3%)	5 (3.6%)	
